# Determining Immune and miRNA Biomarkers Related to Respiratory Syncytial Virus (RSV) Vaccine Types

**DOI:** 10.3389/fimmu.2019.02323

**Published:** 2019-10-09

**Authors:** Lydia J. Atherton, Patricia A. Jorquera, Abhijeet A. Bakre, Ralph A. Tripp

**Affiliations:** Department of Infectious Diseases, College of Veterinary Medicine, University of Georgia, Athens, GA, United States

**Keywords:** RSV, miR, vaccines, immune, disease, microRNA

## Abstract

Respiratory Syncytial Virus (RSV) causes serious respiratory tract illness and substantial morbidity and some mortality in populations at the extremes of age, i.e., infants, young children, and the elderly. To date, RSV vaccine development has been unsuccessful, a feature linked to the lack of biomarkers available to assess the safety and efficacy of RSV vaccine candidates. We examined microRNAs (miR) as potential biomarkers for different types of RSV vaccine candidates. In this study, mice were vaccinated with a live attenuated RSV candidate that lacks the small hydrophobic (SH) and attachment (G) proteins (CP52), an RSV G protein microparticle (GA2-MP) vaccine, a formalin-inactivated RSV (FI-RSV) vaccine or were mock-treated. Several immunological endpoints and miR expression profiles were determined in mouse serum and bronchoalveolar lavage (BAL) following vaccine priming, boost, and RSV challenge. We identified miRs that were linked with immunological parameters of disease and protection. We show that miRs are potential biomarkers providing valuable insights for vaccine development.

## Introduction

Respiratory Syncytial Virus (RSV) is a cause of lower respiratory tract infection (LRTI) worldwide and is responsible for >30 million new LRTI episodes and up to 199,000 deaths in children under 5 years old resulting in more than 3.4 million hospital admission associated with severe RSV disease (1, 2). The elderly population is also markedly affected by RSV (3). Currently, the only approved RSV prophylactic is palivizumab which is used for high-risk patients, but such treatment has limited applicability due to cost and treatment logistics (4–9). Unfortunately, all efforts to develop a safe and effective RSV vaccine have been unsuccessful (10–16). Attempts in the 1960s to develop a formalin-inactivated RSV (FI-RSV) vaccine candidate were hampered by several factors, including lack of protection against RSV infection in infants and young children, and an association with vaccine enhanced disease that resulted in two deaths upon natural RSV infection of vaccinees (10, 17–19). Efforts to develop live attenuated RSV vaccine candidates using cold-passaging, chemical mutagenesis, or reverse genetics have also been unsuccessful largely due to over- or under-attenuation, which currently cannot be precisely predicted (20–30), and natural RSV infection does not provide long-term protective immunity. Several other RSV vaccine platforms have been developed including subunit (31–38), vectored (39–46), particle-based (47–57), or nucleic acid-based (58–63), but none are FDA-approved a feature linked to our incomplete understanding of the host immune response to RSV (10). There are several target populations for RSV vaccines: infants, young children, pregnant women, and the elderly (10). Due to the differences in these target populations, vaccine safety, efficacy, and platform strategies will need to be different (10, 11, 18, 33). By establishing measures of vaccine protection and disease, a wide range of promising vaccine candidates can be evaluated early in development.

The host immune response is important in the outcome of RSV infections (14, 60, 64, 65), and an imbalance between Th1- and Th2-type cytokines is understood to be responsible for a variety of inflammatory disorders (66, 67). Biomarkers can be surrogates for clinical endpoints and are needed to improve vaccine design and efficacy. Small regulatory microRNAs (miRs) have fundamental roles in regulating the expression and function of key immunological mediators such as cytokines (68–70). miR expression profiles have been identified and shown to be useful predictors for several allergic inflammatory diseases (71–74). In addition, specific miRs have been shown to function in regulating key pathogenic mechanisms in asthma and airway hyperresponsiveness, including polarization of adaptive immune responses, activation of T cells (75–78), regulation of eosinophil development (79–84) and modulation of cytokine-driven responses (68–70). miRs are stable, in a variety of tissues, bodily fluids, and sera allowing for sensitive and accurate measurements regarding the physiological state of the individual (72–74). miRs govern host gene expression by inducing mRNA degradation or translation inhibition and have a prominent role in determining the level of protein expression of host gene targets (85–90). Several miRs can also upregulate target gene expression via regulation of promoter function (91, 92). It has been shown that miR patterns of expression vary for numerous physiological processes that have been deemed useful for diagnosis of neurodegenerative disorders, autoimmune diseases, cardiovascular disease, and cancers; likewise miRs have also been implicated in infectious diseases (42, 93–98). Assessing circulating miRs in the sera of patients has supported miR profiling as a powerful non-invasive biomarker tool.

Previously, it was shown that RSV infection of normal tracheal epithelial cells (NTECs) with GFP-expressing RSV (rgRSV) downregulated the expression of multiple miRNAs (99). Of the 24 miRNAs, miR-221 was shown to regulate nerve growth factor (NGF), a key neurokinin that prevents apoptosis in respiratory cells (99). Later, RSV infection of type II respiratory epithelial cells was shown to induce expression of five and down-regulation of three microRNAs via an RSV G protein regulated mechanism (100). RSV deregulated miRNAs were demonstrated to regulate several key immunological pathways. In a follow-up study, RSV infection of normal human bronchoepithelial (NHBE) cells, miRNA deregulation was tied to mechanisms involving IFN beta and the transcription factor NF-κβ (101). We showed that RSV G and NS1/NS2 proteins can modulate miRNA expression (102, 103). Several studies have investigated differential expression of miRNAs in clinical RSV infections and shown deregulated patterns that can be used as potential biomarkers of infections (104–109). While these data show miRNA deregulation during infection, miRNA expression following vaccination with different RSV vaccine candidates under investigation is not well-understood and has the potential to identify safe vs. unsafe vaccine candidates.

As miRs regulate host gene responses, it is important to determine if miR profiles serve to predict safe, efficacious, or diseased vaccine outcomes, particularly since RSV lacks a licensed vaccine. To determine if patterns of miR expression may serve as a surrogate of RSV immunity or disease requires proof of biological relevance. Therefore, we identified miR biomarkers and immune correlates associated with RSV vaccination to establish baselines for biomarker expression across different vaccination types and strategies. Since serum miR profiles provide indications of how miRs may regulate the immune response induced by RSV vaccination or infection (50), serum miR profiles may also suggest vaccine disease outcomes. We hypothesized that RSV infection or vaccination would alter the pattern and tempo of miR expression and that this would be reflected in changes by the host immune or disease response. In the present study, we examined serum miRs in BALB/c mice at various time-points post-RSV vaccination or RSV challenge using several RSV vaccine types. A miR PCR array was used to identify miRs post-vaccination, post-boost, or post-RSV challenge, and correlated with immune parameters and markers of disease.

## Materials and Methods

### Mice

Specific-pathogen-free, 6-to-8 weeks old female BALB/c mice (The Jackson Laboratory) were used. Mice were maintained in microisolator cages with sterilized water and food *ad libitum*. All experiments were approved by and performed in accordance with the guidelines of the University of Georgia Institutional Animal Care and Use Committee (IACUC).

### Viruses and Cell Culture

CP52 was a gift from Stephen Whitehead and Brian Murphy at LID, National Institute of Allergy and Infectious Diseases, Bethesda, MD. CP52 is a cold-passaged live attenuated vaccine strain that lacks the RSV G and SH genes and is derived from RSV B1. RSV A2 and CP52 were propagated in mycoplasma-free Vero E6 cells (ATCC CRL-1586) using DMEM (Gibco) containing 5% FBS (Hyclone) at 37°C/5% CO_2_ and 32°C/5% CO_2_, respectively (110). Viral titers were determined by plaque assay on Vero E6 cells, and plaques were enumerated by an anti-F protein (clone 131-2A) immunostaining assay (111, 112). Infections were performed in serum-free DMEM (SF-DMEM).

### FI-RSV Preparation

The preparation of formalin-inactivated RSV (FI-RSV) vaccine was adapted from the FI-RSV Lot 100 method (113). Briefly, strain A2 was used to infect Vero E6 cells (MOI = 0.1), and at day 4 pi, the cells were lysed following scraping, sonicated, and clarified by centrifugation at 600 x g for 15 min at 4°C. The supernatant was transferred to a tube and filter sterilized using a 2 μm filter; the final protein concentration (determined by BCA) was adjusted to 1 mg/ml. Viral stocks were inactivated by the addition of 37% formalin (final dilution 1:4,000) and incubated at 37°C for 3 days in agitation. FI-RSV was pelleted by ultracentrifugation for 2 h at 25,000 rpm, re-suspended in SF-DMEM at 1/25th of the original volume and adsorbed overnight at room temperature in 4 mg/mL aluminum hydroxide. The compound material was pelleted by centrifugation and the pellet was suspended in SF-DMEM and total virus inactivation was confirmed via plaque assay on Vero cells. This procedure resulted in an FI-RSV vaccine that is concentrated 100-fold and contains 16 mg/ml alum. The vaccine was aliquoted in 1 ml volumes and stored at 4°C.

### RSV GA2 Microparticle-Based Vaccine

A microparticle-based RSV G protein vaccine consisting of 3 μm CaCO_3_ cores was prepared using alternating poly-I-glutamic acid (PGA, negative charge) and poly-I-lysine (PLL, positive charge) layering to build up to seven layers with an RSV G peptide CX3C motif linked to a cationic sequence added as the outermost layer (4). The composition of the seven-layer film was determined using amino acid analysis, which showed that a comparable amount of the peptide component was present in each vaccine batch. Endotoxin levels by limulus amebocyte lysate (LAL) assay were <0.1 EU/μg. The dispersity of the particle vaccines was monitored by dynamic light scattering (DLS). DLS is used to determine the size distribution profile of small particles in suspension or polymers in solution. This layer-by-layer microparticle vaccine has an apparent diameter of ~150 nm for uncoated particles to about 400–500 nm for fully coated particles. Some particle aggregation was detected in each batch with a second population of particles in the 1,500–2,000 nm range.

### Vaccine Delivery

We examined three vaccine types: (1) live-attenuated (CP52), (2) inactivated (FI-RSV), and (3) an RSV G peptide microparticle-based (GA2-MP). The GA2-MP vaccines were suspended in PBS and dispersed by water bath sonication immediately prior to immunization. Doses were adjusted to deliver 50 μg designed peptide (DP)/100 μl/mouse. Mice were subcutaneously (s.c.) immunized with GA2-MP without adjuvant between the shoulder blades. 10^6^ PFU equivalents of FI-RSV was used to intramuscularly (i.m.) vaccinate mice. Mice received a 1:25 dilution of FI-RSV in PBS by i.m. injection in a final volume of 50 μL/mouse. FI-RSV was a positive control for vaccine enhanced disease. 10^6^ PFU of live CP52 diluted in PBS was used to vaccinate mice by intranasal (i.n.) instillation in a final volume of 50 μL/mouse. CP52 was a positive control for vaccine protection. PBS vaccinated mice received 50 μL of PBS (vehicle control) by s.c. injection. Mice were anesthetized by i.p. administration of 2,2,2- tribromoethanol (Avertin; 200 μg/kg Sigma) and a portion of vaccinated mice were i.n. challenged with 10^6^ PFU A2 diluted in PBS.

### Lung Virus Titers and Disease Endpoints

Lung virus titers were determined in treatment and control mice by plaque assay on Vero E6 cells (111). Briefly, lungs were aseptically removed from mice at day 5 post-RSV (10^6^ PFU/mouse) challenge, and individual lung specimens were homogenized at 4°C in 1 mL of SF-DMEM using a gentleMACS™ Dissociator (Miltenyi Biotec). Samples were clarified by centrifugation for 10 min at 200 × g and supernatants were transferred and stored at −80°C. For the plaque assay, 10-fold serial dilutions of the lung homogenates were adsorbed to 90% confluent Vero E6 cell monolayers for 2 h, at 37°C, overlaid with 1% methylcellulose medium and incubated at 37°C for 5 days. RSV plaques were enumerated by immunostaining with monoclonal antibodies against RSV F protein (clone 131-2A) as previously described (112). Lungs from vaccinated and challenged mice were examined for disease pathogenesis, and as anticipated (54, 114, 115), only the lungs from FI-RSV vaccinated mice challenged with RSV showed substantially enhanced disease (data not shown).

### Microneutralization Assay

Two-fold serial dilutions (1:50-1:1,600) of mouse serum in SF-DMEM were incubated with 10^5^ PFU of A2 for 1 h at 37°C, 5% CO_2_. Palivizumab (MedImmune) was used as positive control for neutralizing activity, and positive control wells of virus without sera and negative control wells without virus or sera were included in triplicate on each plate. The antibody-virus mixtures were transferred to 80–90% confluent monolayers of Vero E6 cells in 96-well-plates and incubated for 2 h at 37°C, 5% CO_2_. The virus overlay was aspirated, and 150 μl/well of DMEM-10% FBS was added and plates were incubated for 3–4 days at 37°C, 5% CO_2_, and the plates were fixed with cold 80% acetone in PBS for 10 min, rinsed twice with PBS followed by three washes with 150 μl/well of wash buffer (PBS + 0.1% Tween-20). A monoclonal antibody to the RSV F protein (clone 131-2A) was diluted in PBS with 0.5% gelatin + 0.15% Tween 20 and incubated for 1 h at 37°C, 5% CO_2._ RSV plaques were enumerated using horseradish peroxidase (HRP) conjugated goat anti-mouse IgG (Southern Biotech), developed using TMB substrate (Sigma), and absorbance measured at 450/650 nm dual-wavelength (BioTek Epoch™ microplate spectrophotometer) and Gen5 Data Analysis software. The percentage of neutralization was calculated, and all samples were normalized to the average value from the no serum control wells.

### Indirect ELISA

RSV A2-specific and B1-specific IgG antibodies were detected by ELISA using 96-well-high binding plates (Corning) coated with 10^6^ PFU/mL A2 or B1 in 0.05 M carbonate-bicarbonate buffer, pH 9.6. Sera were added to plates in serial dilutions. RSV-specific antibodies were detected with HRP-conjugated antibodies specific for mouse IgG (Southern Biotech) followed by the addition of SureBlue TMB-peroxidase substrate (KPL, Inc.) for 15 min. Antibody titers were determined as the last sample dilution that generated an OD450 reading of >0.2 (mean OD value of background plus 2 standard deviations of the mean).

### ELISPOT Analysis

MultiScreen filter 96-well-plates (Millipore) were coated with the anti-mouse IL4 or anti-mouse IFNγ capture antibody (R&D Systems) and incubated overnight at 4°C. The plates were then blocked with 200 μL of RPMI-10 medium (RPMI 1640 supplemented with 10% FBS, 100 U/mL penicillin, 100 μg/mL streptomycin, 50 μM 2-mercaptoethanol, and 2 mM L-glutamine) and incubated for 2 h at 37°C. In parallel, spleens were harvested from mice at day 5 post-A2 challenge and prepared to a single cell suspension. The cell suspensions were collected by centrifugation for 10 min at 200 × g and suspended in RPMI-10 at 10^7^ cells/mL. Spleen cell suspensions were added to the wells, and cells were stimulated with either 10 μg/mL RSV M2 _(82−90)_ peptide, 10 μg/mL RSV F _(51−66)_ peptide, 10 μg/mL RSV G _(183−198)_ peptide, or 10 μg/mL eGFP _(200−208)_ (irrelevant peptide control) for 24 h at 37°C and 5% CO_2_. Plates were washed 4 times with wash buffer (0.05% Tween-20 in PBS), anti-mouse IL4 or anti-mouse IFNγ detection antibody (R&D Systems) was added, and plates were incubated overnight at 4°C. Detection antibody was removed, plates were washed, and cytokine spots were developed using NBT/BCIP substrate (R&D Systems). Spots were enumerated using an ELISPOT reader (AID, San Diego).

### Quantification of Cytokines

At day 3 post-A2 challenge, a subset of mice from each group was sacrificed and BAL and sera were collected. The mouse lungs were flushed three times with 1 ml of PBS and the retained BAL was centrifuged at 400 × g for 5 min at 4°C. The recovered supernatants were collected and stored at −80°C until assessed for cytokine concentration, and the cell pellet was suspended in 200 μL of FACS staining buffer (PBS containing 1% BSA). Total cell numbers were counted using a hemocytometer. The Luminex^®^ xMAP system using a MILLIPLEX MAP mouse cytokine immunoassay (Millipore) was used to quantitate cytokines in cell-free BAL supernatants and sera according to the manufacturer protocol. Briefly, beads coupled with anti-IFNγ, anti-IL1α, anti-IL2, anti-IL4, anti-IL5, anti-IL6, anti-IL9, anti-IL10, anti-IL12p40, anti-IL13, anti-IL15, anti-IL17A, anti-MCP1, anti-RANTES, anti-TNFα, and anti-eotaxin monoclonal antibodies were sonicated, mixed, and diluted 1:50 in assay buffer. For the assay, 25 μL of beads were mixed with 25 μL of PBS (for BAL samples) or serum matrix (for serum samples), 25 μL of assay buffer and 25 μL of BAL supernatant or serum and incubated overnight at 4°C. After washing, beads were incubated with biotinylated detection antibodies for 1 h and the reaction mixture was then incubated with streptavidin-phycoerythrin (PE) conjugate for 30 min at room temperature, washed, and suspended in PBS. The assay was analyzed on a Luminex 200 instrument (Luminex Corporation, Austin, TX) using Luminex xPONENT 3.1 software.

### RNA Isolation

Blood was collected from mice via axillary vessels in 1.5 ml microcentrifuge tubes (Fisher), allowed to clot for 30 min at room temperature, and centrifuged at 900 × g for 10 min and 4°C. Serum layer was transferred to a new microcentrifuge tube and centrifuged for 10 min at 16,000 × g and 4°C, and the cleared supernatant was transferred to a new microcentrifuge tube without disturbing the pellet. One hundred microliter of serum sample per mouse was processed for RNA isolation using miRNeasy Serum/Plasma Kit (Qiagen) as per manufacturer's recommended protocol or stored at −80 till processing. Serum/Plasma *C. elegans* miR-39 Spike-In Control (Qiagen) was spiked into each sample prior to RNA purification as an internal control for miR expression profiling in serum to allow for monitoring of RNA recovery and purity, and reverse transcription efficiency. The RNA concentration and purity were determined by Qubit RNA assay broad range (Qubit RNA assay BR, Invitrogen) fluorometry. This reagent specifically binds to RNA only and does not detect DNA, protein or free nucleotides. Additionally, spectrophotometric analysis of all samples using Epoch Gen 5 spectrophotometer (Biotek) showed that all RNA samples had A260/280 ratios ≥1.8.

### miR PCR Arrays and Data Analysis

First-strand cDNA synthesis was performe4d with 200 ng/total RNA from each sample using the miScript II RT kit with miScript HiSpec Buffer (Qiagen) following manufacturer protocol. Briefly, cDNA synthesis was performed at 37°C for 60 min followed by inactivation at 95°C for 5 min. First-strand cDNA was diluted 1:10 in molecular grade water and expression of 84 miRs having a role in T or B cell function was assessed using a miScript miR PCR Array Mouse T cell and B cell Activation (Qiagen) array following the manufacturer's protocol on a Stratagene Mx3000P/3005P real-time instrument. Each array plate contains oligos specific to 84 mature miRs validated to regulate T cell or B cell development and function as well as oligos for spike in (*C. elegans* miR-39), six housekeeping genes [small nucleolar/nuclear RNA (snoRNAs) SNORD61, SNORD95, SNORD96A, SNORD68, SNORD72, and RNU6B] and positive and negative controls for reverse transcription and PCR. Data obtained were analyzed with miScript miR PCR analysis template (Qiagen) using the ΔΔC_T_ method (116). miRs with fold change ≥2 were considered upregulated while miRs with fold change ≤ 0.5 were considered downregulated. RT-qPCR using miR-specific primers was then performed on differentially expressed miRs between treatment groups using a PCR array. The specificity of amplification was validated by dissociation curve analysis.

### Statistical Analysis

All statistical analyses were performed using GraphPad Prism (ver 5.0; GraphPad). Statistical significance was determined using a one-way ANOVA or two-way ANOVA followed by Bonferroni's *post-hoc* comparisons tests; a *p* ≤ 0.05 was considered significant.

### Pathway Analysis

Analysis of pathways regulated by differentially expressed miRs was carried out using DIANA miRPath ver 3.0 (117–119) using the microT-CDS database. The significance of pathway association was determined using a *p*-value threshold ≤ 0.05 and microT threshold of 0.8 using Fisher's exact test. When multiple miRs were analyzed together, data were filtered to identify pathway intersections instead of unions to identify common core pathways regulated by the miRs. Pathway hits were corrected for false discovery rate.

## Results

**Figure 1:**
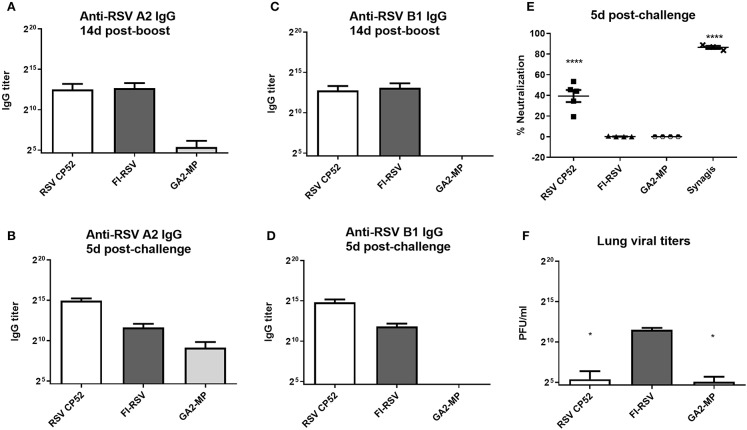
RSV vaccines types, serum IgG, and virus clearance. Sera at day 14 **(A)** and 5 **(B)** post-RSV A or B challenge of prime-boosted mice **(C,D)**; IgG reactivity was determined against A2 **(A,C)** and B1 **(B,D)**. Three weeks after the boost-vaccination mice were i.n. challenged with 10^6^ PFU of A2. **(E)** RSV neutralizing antibody levels were measured by microneutralization assay at day 5 post-RSV challenge. **(F)** Lung virus titers were determined 5 days post-challenge by plaque assay. PBS only-treated groups treated had no detectable effect and are not included. All samples were assayed in duplicate and *n* = 4 mice/group. Error bars represent the SEM and results were considered significant with a ^*^p ≤ 0.05 and ^****^p ≤ 0.0001 as determined by one-way ANOVA and Bonferroni's test.

To assess the serum miR profiles in RSV-vaccinated or challenged mice, the mice were vaccinated (primed) with a live attenuated vaccine (CP52), an inactivated vaccine (FI-RSV), or a microparticle peptide-based vaccine carrying the G CX3C motif (GA2-MP) and boosted 3 weeks later. Three weeks post-boost vaccinated mice were i.n. challenged with 10^6^ PFU A2, and the sera and lung tissues were collected from vaccinated and mock-treated mice at several time-points. Antibody responses to RSV were assessed to confirm that antigens induced a recall response upon vaccination and challenge. RSV A2 or B1-specific serum IgG was determined at 2 weeks post-boost and 5 days post-RSV challenge, and the levels of neutralizing antibody determined at day 5 pi. Lung viral titers were determined at 5 days post-challenge. Broadly, vaccination with CP52 or FI-RSV elicited a cross-reactive humoral response to either A2 or B1 relative to mock (Figures 1A,C, respectively). CP52 (but not FI-RSV) vaccination followed by A2 challenge elicited a strong antibody response (Figures 1B,D, respectively), which was neutralizing (Figure 1E) and correlated with a statistically significant (*p* ≤ 0.05) reduction in lung viral titers (Figure 1F). Despite the G protein CX3C motif having intra-strain conservation (120), it was less immunogenic as indicated by the lower anti-A2 and B1 IgG serum levels. These data show the prototypical responses associated with CP52, GA2-MP, and FI-RSV vaccination in mice (121, 122).

### RSV Vaccine Types and the Th1- and Th2-Type Response

**Figure 2:**
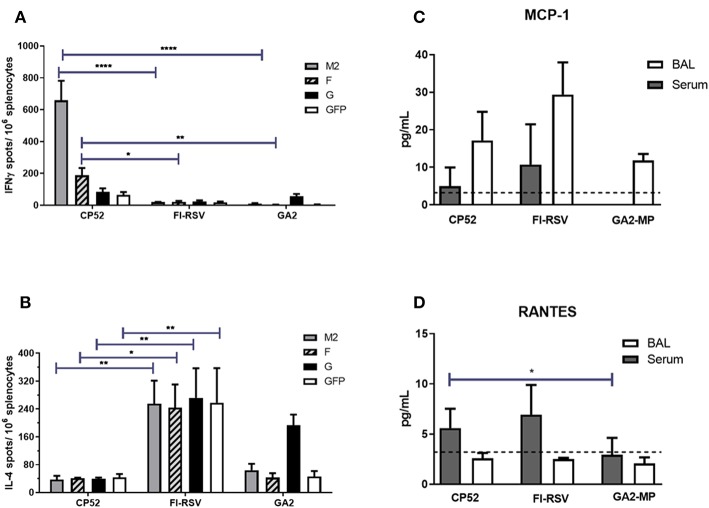
Vaccine types and Th1/ Th2 memory responses. The number of M2_82−90_, F_51−66_, G_183−198_, and eGFP_200−208_-specific (irrelevant peptide control) IL4- and IFNγ- producing splenocytes were determined by ELISPOT harvested at 14 days post-boost vaccination. **(A)** IFNγ- producing splenocytes and **(B)** IL4-producing splenocytes. The data are presented as ELISPOTS/10^6^ splenocytes. Three weeks after the boost mice were i.n. challenged with 10^6^ PFU of A2. The level of **(C)** MCP1 and **(D)** RANTES were measured in sera and BAL supernatant by multiplex cytokine/chemokine assay and the data are presented as pg/mL of cytokine in BAL supernatant at day 3 post-challenge (*n* = 4–6 mice/group). The dashed line indicates the limit of detection (LOD) = 3.2 pg/ml. Error bars represent the SEM from *n* = 4 mice/group and results were considered significant with a ^*^p ≤ 0.05, ^**^p ≤ 0.01, and ^****^p ≤ 0.0001 as determined by two-way ANOVA and Bonferroni's test using GraphPad Prism ver. 8.0.

Th1- or Th2-type responses were assessed by ELISPOT assays, and levels of IFNγ or IL4 expression (representing Th1- or Th2-type responses, respectively) were evaluated at day 14 post-boost vaccination by re-stimulated splenocytes with RSV M2, F or G peptides. As expected, CP52 vaccinated mice had the highest frequency of IFNγ expressing cells compared to IL4 expressing cells (Figure 2A), while splenocytes from FI-RSV vaccinated mice had the highest frequency of IL4 expressing cells compared to IFNγ expressing cells (Figure 2B). GA2-MP vaccinated mice had higher levels of G protein-specific IL4 secreting cells than IFNγ expressing cells, however this difference was not statically significant (*p* > 0.005). MCP1 and RANTES are chemokines involved in leukocyte recruitment to the airway, and to sites of inflammation in response to RSV infection (123–125). Given the role of these immune cell types in disease pathogenesis, levels of MCP1 (Figure 2C) and RANTES (Figure 2D) in sera and BAL were determined by multiplex cytokine/chemokine assays. MCP1 was localized to the lungs, with the highest level in the BAL from FI-RSV vaccinated mice although the level of expression was not substantially different between CP52 and GA2-MP vaccinated mice. In contrast, RANTES was expressed systemically, as evident by a higher level in the sera compared to BAL, with higher levels for FI-RSV and CP52 immune mice compared to GA2-MP vaccinated mice at day 3 post-RSV challenge. As anticipated, no detectable cytokine expression occurred following mock (PBS) treatment (data not shown). These data indicate that CP52 or FI-RSV vaccinated mice have an overall higher level of inflammation than mice vaccinated with GA2-MP upon RSV challenge. Other Th2-specific cytokines (IL4, IL5, IL6, IL10, and IL3) were higher in the sera (data not shown) and BAL of FI-RSV vaccinated mice compared to all other vaccinated groups which further confirm the biased Th2-type cytokine response associated with FI-RSV vaccination.

### The Type of Vaccination Is Linked With Different miR Expression Patterns

Evaluating the Th1- or Th2-type cytokine response using accompanying assays is not efficient for testing of multiple vaccine candidates; however, the examination of miR biomarkers as correlates of the host immune response may aid vetting of safe or disease vaccine types, and considerably accelerate RSV vaccine research. The pattern of miRs can be readily evaluated using PCR (126) in a variety of fluids and tissues (127–133), there is sequence conservation across species (134), and miRs regulate key immunological processes (135). These features can be used to determine baseline data that may differentiate safe from disease risk vaccine types to aid the development of vaccine candidates. Since the memory T cell response is pivotal to development of immunity and disease, we analyzed 84 key miRs connected with T cell function in the sera from vaccinated mice, pre- and post-RSV A2 challenge, at several time-points, e.g., 1-week post-prime/boost or 3d post-challenge. Total RNA was isolated from sera, used for cDNA synthesis, and miR expression was assayed using optimized primer-probes. Fold-changes in miR expression was plotted after normalization.

**Figure 3:**
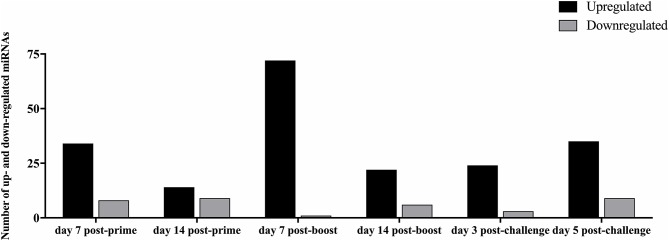
The number of differentially expressed miRNAs during vaccination and post-RSV challenge. Sera miRNA profiles of vaccinated mice (*n* = 4/group) were evaluated at day 7 post-prime, day 14 post-prime, day 7 post-boost, day 14 post-boost, day 3 post-challenge, and day 5 post-challenge using a miRNA PCR array. The y-axis indicates the number of differentially expressed miRNAs. Significance was determined using a fold-change threshold of >2, the result was reported as a fold-upregulation. If the fold change was <0.5, the result was reported as a fold-downregulation.

Analysis of the 84 miRs across all treatments identified miR expression signatures unique to prime-boost vaccination and RSV challenged mice, and those miR signatures conserved among all treatments (Figure 3). In general, each vaccine type, i.e., CP52, FI-RSV, or GA2-MP induced temporal and vaccine-specific miR expression patterns (Figure 3) where miR expression levels were heightened post-boost relative to prime and challenge. Given the differences in vaccine type and vaccination routes, differences were expected and emphasize miR patterns for their utility as potential vaccination biomarkers. Analogously, miR responses following A2 challenge of CP52 vaccinated mice compared to FI-RSV vaccinated mice correlated with safe vs. disease phenotypes of Th1- vs. Th2-type cytokines. Intergroup comparisons identified 58, 70 and 65 miRs differentially expressed in sera from CP52, FI-RSV, and GA2-MP vaccinated mice, respectively. Within groups, 8, 18, and 11 miRs were conserved between prime, boost and 3 days post-challenge for CP52, FI-RSV, and GA2-MP vaccinated mice, respectively. Of the miRs examined, let-7a-5p expression was upregulated >2SD in all vaccinated mice, thus this is likely not a biomarker for distinguishing vaccine-specific responses, but instead a general inflammatory biomarker (Table 1).

**Table 1:** Differentially expressed miRs expressed by the various vaccines.

**Vaccine type**	**Number of miRs**	**Conserved miRs**
GA2-MP	3	miR-26b-5p, miR-346-5p, miR-142a-5p
FI-RSV	9	miR-31-5p, miR-30c-5p, let-7d-5p, miR-326-3p, miR-93-5p, miR-30e-5p, miR-483-5p, let-7g-5p, miR-106b-5p
FI-RSV and GA2-MP	4	miR-20b-5p, let-7f-5p, miR-103-3p, miR-15a-5p
FI-RSV and CP52	4	miR-20a-5p, miR-195a-5p, miR-17-5p, miR-106a-5p
GA2-MP and CP52	3	miR-467f, miR-182-5p, let-7e-5p
GA2-MP, FI-RSV, and CP52	1	let-7a-5p

*Common and unique differentially expressed miRs for GA2-MP, FI-RSV, and CP52 vaccinated mice conserved amongst post-prime (7 and 14 days), post-boost (7 and 14 days), and post-RSV challenge (3 and 5 days) are shown. miRNA expression levels are normalized by SN1/2/3/4/5/6 expression and n = 4 mice/group*.

CP52 vaccination resulted in lower expression of miR-466f-3p and miR-467b-3p and did not induce any miRs. FI-RSV vaccination repressed miR-365-3p and miR-762 expression post-priming, but led to >2.0-fold induction of multiple miRs e.g., let-7d-5p, miR-326-3p, miR-331-3p, miR-16-5p, miR-103-3p, miR-30a-5p, miR-93-5p, miR-181a-5p, miR-101a-3p, miR-15b-5p, miR-15a-3p, miR-106b-5p, miR-142a-3p, miR-19a-3p, miR-30c-5p, miR-101b-3p, miR-25-3p, miR-31-5p, let-7i-5p, let-7g-5p post-prime (Table 2), and miR-326-3p, miR-145a-3p, miR-466f-3p, miR-24-3p, miR-181a-5p, miR-27a-3p, miR-125b-5p, miR-31-5p, miR-214-3p, miR-466f-5p, miR-365-3p, miR-146b-5p, miR-30c-5p, miR-466h-5p, miR-126a-3p post-boost (Table 3). In contrast, GA2-MP vaccination induced only miRs let-7e-5p and miR-26b-5p post-prime and miR-669f-3p, miR-142a-3p post-boost (Table 3). miR-466f-3p had divergent expression between CP52 and FI-RSV vaccinated mice, while miR-142a-3p showed early induction post-FI-RSV but was induced in GA2-MP vaccinated mice post-boost (Table 3).

**Table 2:** miRs induced by the vaccines at post-prime.

**Time-point**	**Vaccine types**	**Upregulated miRs**	**Downregulated miRs**
Day 7 post-prime	GA2-MP, FI-RSV, and CP52	let-7a-5p, miR-142a-5p, miR-20b-5p	miR-467f
	FI-RSV and!!!break GA2-MP	let-7f-5p, miR-15a-5p, miR-98-5p	None
	FI-RSV and!!!break CP52	miR-106a-5p, miR-195a-5p, miR-30e-5p, miR-20a-5p, miR-17-5p, miR-19b-3p	miR-182-5p, miR-466j,!!!break miR-483-5p
	GA2-MP	let-7e-5p, miR-26b-5p	None
	FI-RSV	let-7d-5p, miR-326-3p, miR-331-3p, miR-16-5p, miR-103-3p, miR-30a-5p, miR-93-5p, miR-181a-5p, miR-101a-3p, miR-15b-5p, miR-15a-3p, miR-106b-5p, miR-142a-3p, miR-19a-3p, miR-30c-5p, miR-101b-3p, miR-25-3p, miR-31-5p, let-7i-5p, let-7g-5p	miR-365-3p, miR-762
	RSV and CP52	None	miR-466f-3p,!!!break miR-467b-3p

*Sera miR profiles of vaccinated mice (n = 4/group) were evaluated at day 7 post-prime using a miR PCR array. The relative expression levels of candidate miRs selected from the PCR array analysis were validated by RT-qPCR. Values are represented as fold-change/mock (PBS vaccinated/RSV A2 challenge). miR levels were normalized by RNU6B gene expression and all samples were run in duplicate. Fold-change was calculated using 2^(−ΔΔCT)^ method. Differential expression was determined using the following criteria, if the fold change was >2, the result was reported as a fold-upregulation. If the fold-change was <0.5, the result was reported as a fold-downregulation*.

**Table 3:** miRs induced by the vaccines at post-boost vaccination.

**Time-point**	**Vaccine type**	**Upregulated-miRs**	**Downregulated-miRs**
Day 7 post-boost	GA2-MP, FI-RSV, and CP52	miR-195a-5p, miR-320-3p, let-7a-5p, miR-181b-5p, miR-672-5p, let-7e-5p, miR-17-5p, let-7c-5p, miR-714, let-7d-5p, let-7f-5p, miR-574-5p, miR-182-5p, miR-16-5p, miR-467f, miR-21a-5p, miR-130b-3p, miR-1187, miR-15b-5p, miR-26b-5p, miR-20a-5p, miR-184-3p, miR-762, miR-20b-5p, miR-25-3p, let-7i-5p, let-7g-5p	None
	FI-RSV and GA2-MP	miR-331-3p, miR-103-3p, miR-29a-3p, miR-30e-5p, miR-23b-3p, miR-101a-3p, miR-106b-5p, miR-142a-5p, miR-19b-3p, miR-19a-3p, miR-101b-3p, miR-30b-5p, miR-221-3p, miR-106a-5p, miR-30a-5p, miR-346-5p, miR-93-5p, miR-29b-3p, miR-466j, miR-15a-3p, miRR-15a-3p, miR-29c-3p	None
	GA2-MP and CP52	miR-223-3p, miR-669e-5p, miR-98-5p, miR-26a-5p, miR-155-5p	None
	FI-RSV and CP52	miR-483-5p, miR-1196-5p	
	GA2-MP	miR-669f-3p, miR-142a-3p	None
	FI-RSV	miR-326-3p, miR-145a-3p, miR-466f-3p, miR-24-3p, miR-181a-5p, miR-27a-3p, miR-125b-5p, miR-31-5p, miR-214-3p, miR-466f-5p, miR-365-3p, miR-146b-5p, miR-30c-5p, miR-466h-5p, miR-126a-3p	None

*Sera miR profiles of vaccinated mice (n = 4/group) were evaluated at day 7 post-boost using a miR PCR array. The relative expression levels of candidate miRs selected from the PCR array analysis were validated by RT-qPCR. Values are represented as fold-change/mock (PBS vaccinated/RSV A2 challenge). miR levels were normalized by RNU6B gene expression and all samples were run in duplicate. Fold-change was calculated using 2^(−ΔΔCT)^ method. Differential expression was determined using the following criteria, if the fold change was >2, the result was reported as a fold upregulation. If the fold-change was <0.5, the result was reported as a fold-downregulation*.

miR profiling for each vaccine type post-challenge showed unique patterns and tempos of expression. For example, CP52 vaccinated mice had higher expression of let-7f-5p, miR-103-3p, miR-15b-5p, miR-101a-3p, miR-16-5p, miR-20a-5p, miR-106a-5p, miR-98-5p, miR-30a-5p, miR-17-5p, miR-195a-5p, miR-142a-5p, miR-181a-5p, miR-714, miR-31-5p, miR-101b-3p, miR-25-3p, let-7i-5p, miR-130b-3p, and reduced miR-182-5p post-challenge (Table 4). In contrast, FI-RSV vaccinated mice showed repression of miR-483-5p post-challenge, while GA2-MP vaccinated mice had induction of miR-145a-5p, miR-346-5p, miR-146b-5p, and repression of miR-669e-5p post-challenge. Thus, comparing between vaccine groups at 7 days post-prime, 7 days post-boost, or 3 days post-RSV challenge showed miRs profiles as related to safe or disease responses (Table 5).

**Table 4:** miRs at post-RSV challenge.

**Time-point**	**Vaccine type**	**Upregulated-miRs**	**Downregulated-miRs**
Day 3 post-challenge	GA2-MP and RSV CP52	miR-467f, miR-184-3p	None
	GA2-MP	miR-145a-5p, miR-346-5p, miR-146b-5p	miR-669e-5p
	FI-RSV	None	miR-483-5p
	CP52	let-7f-5p, miR-103-3p, miR-15b-5p, miR-101a-3p, miR-16-5p, miR-20a-5p, miR-106a-5p, miR-98-5p, miR-30a-5p, miR-17-5p, miR-195a-5p, miR-142a-5p, miR-181a-5p, miR-714, miR-31-5p, miR-101b-3p, miR-25-3p, let-7i-5p, miR-130b-3p	miR-182-5p

*Sera miR profiles of vaccinated mice (n = 4/group) were evaluated at day 3 post-challenge using a miR PCR array. The relative expression levels of candidate miRs selected from the PCR array analysis were validated by RT-qPCR. Values are represented as fold-change/mock (PBS vaccinated/RSV A2 challenge). miR levels were normalized by RNU6B gene expression and all samples were run in duplicate. Fold-change was calculated using 2^(−ΔΔCT)^ method. Differential expression was determined using the following criteria, if the fold change was >2, the result was reported as a fold-upregulation. If the fold change was <0.5, the result was reported as a fold-downregulation*.

**Table 5:** Patterns of miR expression following prime, boost, and challenge.

	**Fold-change**					
	**Prime**		**Boost**		**Challenge (day 3)**	
	**>2.0**	**≤0.5**	**>2.0**	**≤0.5**	**>2.0**	**≤0.5**
CP52		**miR-466f-3p**, miR-467b-3p		NA	let-7f-5p, miR-103-3p, miR-15b-5p, miR-101a-3p, miR-16-5p, miR-20a-5p, miR-106a-5p, miR-98-5p, miR-30a-5p, miR-17-5p, miR-195a-5p, miR-142a-5p, miR-181a-5p, miR-714, miR-31-5p, miR-101b-3p, miR-25-3p, let-7i-5p, miR-130b-3p	miR-182-5p
FI-RSV	Let-7d-5p, miR-326-3p, miR-331-3p, miR-16-5p, miR-103-3p, miR-30a-5p, miR-93-5p, miR-181a-5p, miR-101a-3p, miR-15b-5p, miR-15a-3p, miR-106b-5p, **miR-142a-3p**, miR-19a-3p, miR-30c-5p, miR-101b-3p, miR-25-3p, miR-31-5p, let-7i-5p, let-7g-5p	miR-365-3p, miR-762	miR-326-3p, miR-145a-3p, **miR-466f-3p**, miR-24-3p, miR-181a-5p, miR-27a-3p, miR-125b-5p, miR-31-5p, miR-214-3p, miR-466f-5p, miR-365-3p, **miR-146b-5p**, miR-30c-5p, miR-466h-5p, miR-126a-3p	NA		miR-483-5p
GA2-MP	Let-7e-5p, miR-26b-p		miR-669-3p, **miR-142a-3p**	NA	miR-145a-5p, miR-346-5p, **miR-146b-5p**	miR-669e-5p

*miR expression for the top 96 miRs associated with T cell development and function were evaluated in sera obtained day 7 post-prime, day 7 post-boost, or day 3 post-challenge using miR qPCR arrays. Fold-change was calculated using ΔΔCt method relative to several reference genes that showed no change in expression across time-points and treatments. All data represent >3 independent experiments. miRs common between time-points or treatments are highlighted in bold. NA, not applicable*.

### miR Patterns Specific to the Vaccine Type

To determine if serum miR profiles were specific to a vaccine type e.g., live (CP52) i.n. delivered, or killed (FI-RSV) i.m. delivered, or subunit (GA2-MP) s.c. delivered, the miRs were evaluated from vaccinated mice at day 7 post-vaccination. Interestingly, we observed nearly twice as many miRs expressed in the sera at day 7 post-vaccination compared to 14 days post-vaccination (Supplementary Table 1). The sera miR profiles showed that CP52 vaccinated mice had decreased miR-466f-3p and miR-467b-3p expression at 7 days post-vaccination, whereas sera miR expression from FI-RSV-vaccinated mice had two downregulated miRs (miR-365-3p and miR-62) while sera from GA2-MP vaccinated mice expressed higher miRs, i.e., let-7e-5p and miR-26b-5p at 7 days post-prime (Table 2). Interestingly, miR-467f expression was downregulated for all vaccine types at day 7 post-vaccination. Additionally, several miRs were identified in all vaccinated mice, e.g., let-7a-5p, miR-142a-5p, and miR-20b-5p which were upregulated (Table 1). The results showed that several miRs were expressed specifically to CP52 (miR-466f-3p, miR-467b-3p), to FI-RSV (let-7d-5p, miR-326-3p, miR-331-3p, miR-16-5p, miR-103-3p, miR-30a-5p, miR-93-5p, miR-181a-5p, miR-101a-3p, miR-15b-5p, miR-15a-3p, miR-106b-5p, miR-142a-3p, miR-19a-3p, miR-30c-5p, miR-101b-3p, miR-25-3p, miR-31-5p, let-7i-5p, let-7g-5p) and to GA-M2 vaccines types (let-7e-5p, miR-26b-5p).

### miRs Patterns Induced by Post-Boost Vaccination and RSV Challenge

Serum miR profiles were examined to determine the miR profiles by the vaccine types post-boost (Table 3). Of 75 differentially expressed miRs evaluated (Table 3; Figure 3), the miRs commonly expressed were miR-195a-5p, miR-320-3p, let-7a-5p, miR-181b-5p, miR-672-5p, let-7e-5p, miR-17-5p, let-7c-5p, miR-714, let-7d-5p, let-7f-5p, miR-574-5p, miR-182-5p, miR-16-5p, miR-467f, miR-21a-5p, miR-130b-3p, miR-1187, miR-15b-5p, miR-26b-5p, miR-20a-5p, miR-184-3p, miR-762, miR-20b-5p, miR-25-3p, let-7i-5p, let-7g-5p which were induced by all vaccine types. As these miRs are commonly expressed it is likely their expression is linked to a general response, i.e., the pro-inflammatory response associated with vaccination. The serum miRs upregulated specific to CP52 vaccinated mice were miR-98-5p, miR-26a-5p, miR-155-5p, miR-223-3p, miR-669e-5p, for GA2-MP vaccinated mice miR-669f-3p and miR-142a-3p, and fifteen miRs were upregulated in the sera of FI-RSV vaccinated mice. Two miRs, miR-669f-3p and miR-142a-3p, were commonly expressed in CP52 and GA2-MP vaccinated mice, and 50 miRs were differentially expressed post-RSV challenge of vaccinated mice. All data are shown in Supplementary Tables 2, 3. At day 3 post-RSV challenge, 24 miRs were upregulated for all vaccinated mice types compared to mock-vaccinated (Table 4). For CP52-vaccinated mice, several serum miRs were upregulated (e.g., let-7f-5p, miR-103-3p, miR-15b-5p, miR-101a-3p, miR-16-5p, miR-20a-5p, miR-106a-5p, miR-98-5p, miR-30a-5p, miR-17-5p, miR-195a-5p, miR-142a-5p, miR-181a-5p, miR-714, miR-31-5p, miR-101b-3p, miR-25-3p, let-7i-5p, miR-130b-3p), for FI-RSV-vaccinated mice only miR-483-5p was upregulated, and for GA2-MP vaccinated mice miR-145a-5p, miR-346-5p, and miR-146b-5p were upregulated. Interestingly, miR-184-3p was expressed in the sera by all vaccine groups suggesting that this miR is not vaccine-specific. The data from these studies is summarized in Table 5.

### miRs and the Host Pathway

miRs act as rheostats to subtly regulate aspects of the host immune response to virus infection and vaccination (64). They fine-tune responses, adjust functions, and bolster or dampen immune operations to maintain homeostasis. The pattern of miR expression highlights their function, i.e., constrain or enhance responses in a temporal fashion. As this study examines the pattern and tempo of miRs expressed in the response to vaccination and challenge, it is not surprising to identify unique and common miR profiles and those that are differentially expressed during and after vaccination or challenge. A goal of these studies is to determine if the miR expression patterns can be used to predict safe or unsafe responses to vaccination or challenge. Viral infection and vaccination induce inflammation and determining miR pathways that are induced or repressed in mice can help differentiate safe vs. disease risk vaccines. As we examined the miR expression pattern linked to the type of vaccine and the cytokine response to RSV vaccination and challenge, we analyzed the gene pathways that could be regulated by miRs that are induced or repressed following CP52, FI-RSV or GA2-MP priming, prime-boost, and RSV challenge using DIANA miRPath (117–119). The intersecting pathways were selected for examination having a *p*-value cutoff of *p* < 0.05 (Table 5). CP52 vaccination downregulated miR-466f-3p and miR467b-3p which is known to regulate genes of the TGFβ signaling which has been shown to have an important role in RSV replication and inflammation leading to lung injury, fibrosis, and remodeling (136–144). Additionally, these miRs are predicted to regulate cancer-related pathways (Table 6; Supplemental Table 1) which contain many of the top genes involved in cell cycle control, a feature live RSV infection is known to regulate (65, 102, 142, 145–147). FI-RSV priming induced miRs predicted to regulate mucin biosynthesis, axon guidance, and other pathways in cancer (Table 2) while GA2-MP primed miRs were predicted to regulate Lysine degradation, proteoglycan expression and function and FoxO signaling (Table 6). FoxO signaling pathway has been shown to regulate the innate immune pathways in respiratory epithelium following infection (148). Analysis of miR at 7d post-boost showed distinct miR pathway profiles among candidate vaccines. While both CP52 and GA2-MP boosting did not alter miR expression, FI-RSV boosting led to many deregulated miRs (Table 5). In particular, the fatty acid metabolism pathway is predicted to be regulated by these miRs. Fatty acid metabolism is essential for RSV replication (149). Additional pathways predicted to be regulated by miRs and linked to FI-RSV boosting include lysine degradation and steroid biosynthesis (Table 6). GA2-MP boosting affected miRs patterns predicted to regulate adherens junction signaling which are associated to disruption of the airway barrier during infection [Table 7; (150–152)]. miRs deregulated following RSV challenge in CP52 or GA2-MP vaccinated mice were predicted to regulate multiple pathways in fatty acid metabolism and pluripotency, likely related to cell cycle (Table 8). Additionally, TGFb and Hippo signaling pathways were also shown to likely regulated by the miR expression patterns (Table 8). The Hippo pathway is thought to be involved in modulating the potency of anti-viral response particularly in a nutritional deprivation state (153).

**Table 6:** Pathway analysis following priming by different vaccine types.

**Treatment**	**Pathway name**	***p*-value**	**# of miRs**
**Vaccine Type**			
CP52	TGFb signaling pathway	3.55e-12	18
	Endometrial cancer pathway	1.04e-09	11
	Prostate cancer pathway	8.35e-08	12
FI-RSV	Mucin type O glycan biosynthesis	3.63e-13	6
	Axon guidance	9.07e-13	7
	Pathways in cancer	3.99e-11	7
GA2-MP	Lysine degradation	3.09e-14	2
	Proteoglycans in cancer	2.24e-06	2
	FoxO signaling pathways	1.44e-05	2

*Pathways regulated by miRs in Table 1 were analyzed using DIANA miRPath (117–119) using the microT-CDS database. E-values designate statistical confidence ascribed to gene hits for pathways using standard hypergeometric distribution and meta-analysis statistics (p <0.05, Fisher's exact t-test)*.

**Table 7:** Pathway analysis of miRs deregulated following boosting by different vaccine types.

	**Pathway name**	***p*-value**	**# of miRs**
**Post-boost**			
CP52	No deregulated miR	NA	NA
FI-RSV	Fatty acid metabolism	1.33e-13	3
	Lysine degradation	2.13e-10	7
	Steroid biosynthesis	3.99e-06	4
GA2-MP	Adherens junction signaling	0.012	2

*Pathways regulated by miRs in Table 1 were analyzed using DIANA miRPath (117–119) using the microT-CDS database. E-values designate statistical confidence ascribed to gene hits for pathways using standard hypergeometric distribution and meta-analysis statistics (p <0.05, Fisher's exact t-test). NA, not applicable*.

**Table 8:** Pathway analysis of miRs deregulated following challenge of vaccinated mice.

	**Pathway name**	***p*-value**	**# of miRs**
**Post-challenge**			
CP52	Fatty acid metabolism	6.22e-16	15
	Prion diseases	9.06e-09	13
	Fatty acid degradation	3.12e-08	14
FI-RSV	No deregulated miR	NA	NA
GA2-MP	TGFb signaling	6.17e-05	2
	Hippo signaling pathways	0.000474	2
	Signaling pathways regulations pluripotency of stem cells	0.009	2

*Pathways regulated by miRs in Table 1 were analyzed using DIANA miRPath (117–119) using the microT-CDS database. E-values designate statistical confidence ascribed to gene hits for pathways using standard hypergeometric distribution and meta-analysis statistics (p <0.05, Fisher's exact t-test). NA, not applicable*.

The results indicate that the miR profiles and their tempos of expression are adjusted to the type of vaccine and challenge, an effect linked to both non-specific responses (e.g., inflammation) and specific immune responses (e.g., T cell activation or memory). It is important to note that some miRs are unaffected by vaccination while others undergo a global up- or down-regulation upon vaccination or challenge. For reasons of brevity, we have focused on understanding those miR expression patterns induced ≥2SD above the control. It is important to note that several immune regulatory molecules are miR targets. Specifically, cytokines/chemokines are immune effector molecules and are integrated in the net responses to vaccination and challenge. miRs control the activation and integration of the pathways to support T cell responses while maintaining homeostasis. Additional information regarding the miR host pathway analysis can be found in Data Sheet 1 of the Supplementary Material section.

## Discussion

The development of safe and effective RSV vaccine candidates can be assisted by a better understanding of biomarker expression. Biomarkers may allow for the prediction of probable vaccine candidate outcomes. Additional analyses are needed to further aid decisions regarding vaccine candidates, but ways to improve RSV vaccine candidate selection has become paramount after more than 5 decades of unsuccessful research efforts. We hypothesized that assessment of miR profiles with general Th1/Th2 cytokine responses would enable correlations with safe, live vaccines (CP52), subunit vaccines (GA-M2), and disease-enhancing vaccines (FI-RSV) to help develop baselines for a better understanding of prospective RSV vaccine candidates. In this study, show vaccine-specific and temporal miRNA expression profiles relating to efficacy or vaccine-associated disease. We examined miR expression profiles of vaccinated mice pre- and post-RSV challenge were determined for 84 miRs associated with T cell responses and function. We showed that while both CP52 and FI-RSV vaccination induce a humoral response, only CP52 induced a neutralizing antibody response leading to reduction in RSV replication (Figure 1E). Further, splenocytes from CP52, FI-RSV, or GA2-MP vaccinated mice were stimulated with RSV-specific peptides then assayed for Th1-type or Th2-type cytokines. The cytokine and miR expression showed that M2_82−90_ re-stimulation of splenocytes from CP52 but not FI-RSV vaccinated mice led to a strong induction of IFNg which is characteristic of a Th1 response. In contrast, peptide stimulation of splenocytes from FI-RSV vaccinated mice led to a strong induction of IL4, a cytokine characteristic of a Th2-type response. Peptide stimulation of splenocytes from GA2-MP vaccinated mice led to a higher number of G_183−198_ IL4- and IFN-specific secreting cells, characteristic of a balanced Th1-/Th2-type response to the G protein. Taken together, these results led to the assessment that CP52 vaccination primes for a safe response while FI-RSV primes for disease following vaccination and GA2-MP primes for a mostly balanced cytokine response.

The miR PCR array showed differential expression of a conserved set of miRs across prime-boost vaccination and RSV-challenge, more specifically 11 miRNAs in GA2-MP vaccinated mice, 18 miRs in FI-RSV vaccinated mice, and 8 miRs in RSV CP52 vaccinated mice. Several of these miRs have been shown to participate in the regulation of the immune response, and in some cases are associated with RSV infection. FI-RSV vaccinated mice had let-7d-5p, let-7f-5p, and let-7g-5p miR expression at post-prime, post-boost, and post-challenge. GA2-MP vaccinated mice had similar results with let-7e-5p and let-7f expression. Members of the let-7 family target IL-6 expression, and has an extensive list of other experimentally validated targets including SOCS4, caspase-3, p27, TLR4, IL-13, and IL10 (101). Let-7 could be a mechanism of IL-6 regulation during RSV infection (101). RSV infection induces secretion of numerous pro-inflammatory cytokines, including type I and types II IFNs, TNFα, IL-12, and IL-6 (101, 154–156). Mice vaccinated with CP52 or GA2-MP induced differential miR-467f expression during prime-boost vaccination and post-challenge. Previous miR screens for respiratory viruses have not previously identified miR-467f; however, a microarray-based approach to evaluate the miR profile of HIV-associated nephropathy in a mouse model showed that treatment with rapamycin (an mTOR inhibitor) to halt disease progression induced upregulation of miR-467f expression (157). Interestingly, rapamycin inhibits RSV-induced mTOR activation and increases the frequency of RSV-specific CD8 T cells and RSV-specific memory T cell precursors in mice (158). Therefore, miR-467f may have a role in cellular immunity during vaccination and RSV infection. miR-106a-5p and miR-106b-5p expression levels were upregulated in FI-RSV vaccinated mice during prime-boost vaccination and post-challenge with RSV. Interestingly, allergic airway inflammation in mice has been associated with increased miR-106a expression and decreased IL-10, suggesting that miR-106a may regulate IL-10 expression and Th2-type responses (159, 160). Expression levels of miR-30c-5p and miR-30e-5p (from the miR-30/384-5p family) were upregulated in FI-RSV vaccinated mice at prime and boost-vaccination, and post-challenge. Upregulation of miR-30c-5p expression in the airway wall has been shown in a BALB/c mouse model of chronic asthma (161). The miR profiles identified for vaccine-induced protection and vaccine-enhanced disease appear to correlate with protective immune functions and airway inflammation, respectively. Although this study produced a list of miRs that may regulate RSV vaccine efficacy, additional studies are warranted to clarify the mechanisms behind how these miRs mediate host-virus interactions.

Overall, the results from these studies show that vaccine candidates associated safe or disease responses exhibited differential miR profiles following boosting which were higher in magnitude compared to priming or RSV challenge sera specimens. The results demonstrate that a considerable number of miRs are different between vaccine types, and a common set of miRs is expressed for all vaccine treatments. Pathway analysis of miR targets identified pathways correlated with inflammation particularly those that may contribute to airway inflammation, leukocyte recruitment and alveolar infiltration (12, 162, 163). The miR profiles from vaccinated mice were linked to cytokine phenotypes of protection or disease and appear to correlate with miRs that regulate protective immune functions or airway inflammation. Additional studies are warranted to validate miR phenotypes to determine the mechanisms of action linked to host gene regulation, and the associated immune response to determine their value as predictive biomarkers. These studies show that serum miR profiles may offer a proxy to assist vaccine development and facilitate a better understanding of vaccine studies.

## Ethics Statement

Specific-pathogen-free, 6-to-8 weeks old female BALB/c mice (The Jackson Laboratory) were used. Mice were maintained in microisolator cages with sterilized water and food *ad libitum*. All experiments were performed in accordance with the guidelines of the University of Georgia Institutional Animal Care and Use Committee (IACUC).

## Author Contributions

LA, PJ, AB, and RT conceived, designed, performed, analyzed the experiments, and wrote the manuscript. RT contributed reagents, materials, and analysis tools.

### Conflict of Interest

The authors declare that the research was conducted in the absence of any commercial or financial relationships that could be construed as a potential conflict of interest.

## References

[1] Shi T McAllister DA O'Brien KL Simoes EAF Madhi SA Gessner BD. Global, regional, and national disease burden estimates of acute lower respiratory infections due to respiratory syncytial virus in young children in 2015: a systematic review and modelling study. Lancet. (2017) 390:946–58. 10.1016/S0140-6736(17)30938-8 28689664 PMC5592248

[2] Hall CB Weinberg GA Iwane MK Blumkin AK Edwards KM Staat MA. The burden of respiratory syncytial virus infection in young children. N Engl J Med. (2009) 360:588–98. 10.1056/NEJMoa0804877 19196675 PMC4829966

[3] Parikh RC McLaurin KK Margulis AV Mauskopf J Ambrose CS Pavilack M. Chronologic age at hospitalization for respiratory syncytial virus among preterm and term infants in the United States. Infect Dis Ther. (2017) 6:477–86. 10.1007/s40121-017-0167-9 28866800 PMC5700888

[4] Anderson EJ Carosone-Link P Yogev R Yi J Simoes EAF. Effectiveness of palivizumab in high-risk infants and children: a propensity score weighted regression analysis. Pediatr Infect Dis J. (2017) 36:699–704. 10.1097/INF.0000000000001533 28709160 PMC5516669

[5] Hoch HE Collaco JM. Recurrent wheezing in childhood and palivizumab. Am J Respir Crit Care Med. (2017) 196:1–2. 10.1164/rccm.201701-0256ED 28665205

[6] Mochizuki H Kusuda S Okada K Yoshihara S Furuya H Simoes EAF. Palivizumab prophylaxis in preterm infants and subsequent recurrent wheezing. Six-year follow-up study. Am J Respir Crit Care Med. (2017) 196:29–38. 10.1164/rccm.201609-1812OC 28152315

[7] Resch B. Product review on the monoclonal antibody palivizumab for prevention of respiratory syncytial virus infection. Hum Vaccin Immunother. (2017) 13:2138–49. 10.1080/21645515.2017.1337614 28605249 PMC5612471

[8] Sanchez-Luna M Burgos-Pol R Oyaguez I Figueras-Aloy J Sanchez-Solis M Martinon-Torres F. Cost-utility analysis of Palivizumab for Respiratory Syncytial Virus infection prophylaxis in preterm infants: update based on the clinical evidence in Spain. BMC Infect Dis. (2017) 17:687. 10.1186/s12879-017-2803-0 29041909 PMC5645982

[9] Wong SK Li A Lanctot KL Paes B. Adherence and outcomes: a systematic review of palivizumab utilization. Expert Rev Respir Med. (2018) 12:27–42. 10.1080/17476348.2018.1401926 29130355

[10] Anderson LJ Dormitzer PR Nokes DJ Rappuoli R Roca A Graham BS. Strategic priorities for respiratory syncytial virus (RSV) vaccine development. Vaccine. (2013) 31(Suppl. 2):B209–15. 10.1016/j.vaccine.2012.11.106 23598484 PMC3919153

[11] Drysdale SB Sande CJ Green CA Pollard AJ. RSV vaccine use–the missing data. Expert Rev Vaccines. (2016) 15:149–52. 10.1586/14760584.2016.1114419 26636902

[12] Graham BS. Vaccine development for respiratory syncytial virus. Curr Opin Virol. (2017) 23:107–12. 10.1016/j.coviro.2017.03.012 28525878 PMC5653266

[13] Higgins D Trujillo C Keech C. Advances in RSV vaccine research and development - a global agenda. Vaccine. (2016) 34:2870–5. 10.1016/j.vaccine.2016.03.109 27105562

[14] Jorquera PA Anderson L Tripp RA. Understanding respiratory syncytial virus (RSV) vaccine development and aspects of disease pathogenesis. Expert Rev Vaccines. (2016) 15:173–87. 10.1586/14760584.2016.1115353 26641318

[15] Rezaee F Linfield DT Harford TJ Piedimonte G. Ongoing developments in RSV prophylaxis: a clinician's analysis. Curr Opin Virol. (2017) 24:70–8. 10.1016/j.coviro.2017.03.015 28500974 PMC5541395

[16] Roberts JN Graham BS Karron RA Munoz FM Falsey AR Anderson LJ. Challenges and opportunities in RSV vaccine development: meeting report from FDA/NIH workshop. Vaccine. (2016) 34:4843–9. 10.1016/j.vaccine.2016.07.057 27566900

[17] Becker Y. Respiratory syncytial virus (RSV) evades the human adaptive immune system by skewing the Th1/Th2 cytokine balance toward increased levels of Th2 cytokines and IgE, markers of allergy–a review. Virus Genes. (2006) 33:235–52. 10.1007/s11262-006-0064-x 16972040

[18] Castilow EM Olson MR Varga SM. Understanding respiratory syncytial virus (RSV) vaccine-enhanced disease. Immunol Res. (2007) 39:225–39. 10.1007/s12026-007-0071-6 17917067

[19] Johnson TR Graham BS. Contribution of respiratory syncytial virus G antigenicity to vaccine-enhanced illness and the implications for severe disease during primary respiratory syncytial virus infection. Pediatr Infect Dis J. (2004) 23:S46–57. 10.1097/01.inf.0000108192.94692.d2 14730270

[20] Boyoglu-Barnum S Todd SO Meng J Barnum TR Chirkova T Haynes LM. Mutating the CX3C motif in the G protein should make a live respiratory syncytial virus vaccine safer and more effective. J Virol. (2017) 91:e02059–16. 10.1128/JVI.02059-16 28275196 PMC5411601

[21] Connors M Crowe JE Jr Firestone CY Murphy BR Collins PL. A cold-passaged, attenuated strain of human respiratory syncytial virus contains mutations in the F and L genes. Virology. (1995) 208:478–84. 10.1006/viro.1995.1178 7747420

[22] Gonzalez IM Karron RA Eichelberger M Walsh EE Delagarza VW Bennett R. Evaluation of the live attenuated cpts 248/404 RSV vaccine in combination with a subunit RSV vaccine (PFP-2) in healthy young and older adults. Vaccine. (2000) 18:1763–72. 10.1016/S0264-410X(99)00527-7 10699324

[23] Groppo R DiNapoli J Il Jeong K Kishko M Jackson N Kleanthous H. Effect of genetic background and delivery route on the preclinical properties of a live attenuated RSV vaccine. PLoS ONE. (2018) 13:e0199452. 10.1371/journal.pone.0199452 29920563 PMC6007926

[24] Hsu KH Crowe JE Jr Lubeck MD Davis AR Hung PP Chanock RM. Isolation and characterization of a highly attenuated respiratory syncytial virus (RSV) vaccine candidate by mutagenesis of the incompletely attenuated RSV A2 ts-1 NG-1 mutant virus. Vaccine. (1995) 13:509–15. 10.1016/0264-410X(94)00002-5 7543716

[25] Jin H Cheng X Traina-Dorge VL Park HJ Zhou H Soike K. Evaluation of recombinant respiratory syncytial virus gene deletion mutants in African green monkeys for their potential as live attenuated vaccine candidates. Vaccine. (2003) 21:3647–52. 10.1016/S0264-410X(03)00426-2 12922094

[26] Karron RA Luongo C Thumar B Loehr KM Englund JA Collins PL. A gene deletion that up-regulates viral gene expression yields an attenuated RSV vaccine with improved antibody responses in children. Sci Transl Med. (2015) 7:312ra175. 10.1126/scitranslmed.aac8463 26537255 PMC6342448

[27] Le Nouen C Brock LG Luongo C McCarty T Yang L Mehedi M. Attenuation of human respiratory syncytial virus by genome-scale codon-pair deoptimization. Proc Natl Acad Sci USA. (2014) 111:13169–74. 10.1073/pnas.1411290111 25157129 PMC4246931

[28] Malkin E Yogev R Abughali N Sliman J Wang CK Zuo F. Safety and immunogenicity of a live attenuated RSV vaccine in healthy RSV-seronegative children 5 to 24 months of age. PLoS ONE. (2013) 8:e77104. 10.1371/journal.pone.0077104 24204744 PMC3812203

[29] Russell RF McDonald JU Ivanova M Zhong Z Bukreyev A Tregoning JS. Partial attenuation of respiratory syncytial virus with a deletion of a small hydrophobic gene is associated with elevated interleukin-1beta responses. J Virol. (2015) 89:8974–81. 10.1128/JVI.01070-15 26085154 PMC4524082

[30] Wright PF Karron RA Belshe RB Thompson J Crowe JE Jr Boyce TG. Evaluation of a live, cold-passaged, temperature-sensitive, respiratory syncytial virus vaccine candidate in infancy. J Infect Dis. (2000) 182:1331–42. 10.1086/315859 11010838

[31] Blanco JCG Pletneva LM McGinnes-Cullen L Otoa RO Patel MC Fernando LR. Efficacy of a respiratory syncytial virus vaccine candidate in a maternal immunization model. Nat Commun. (2018) 9:1904. 10.1038/s41467-018-04216-6 29765035 PMC5953919

[32] Cayatte C Snell Bennett A Rajani GM Hostetler L Maynard SK Lazzaro M. Inferior immunogenicity and efficacy of respiratory syncytial virus fusion protein-based subunit vaccine candidates in aged versus young mice. PLoS ONE. (2017) 12:e0188708. 10.1371/journal.pone.0188708 29182682 PMC5705161

[33] Esposito S Pietro GD. Respiratory syncytial virus vaccines: an update on those in the immediate pipeline. Future Microbiol. (2016) 11:1479–90. 10.2217/fmb-2016-0106 27750448

[34] Garg R Latimer L Gomis S Gerdts V Potter A van Drunen Littel-van den Hurk S. Maternal vaccination with a novel chimeric glycoprotein formulated with a polymer-based adjuvant provides protection from human parainfluenza virus type 3 in newborn lambs. Antiviral Res. (2019) 162:54–60. 10.1016/j.antiviral.2018.12.010 30550799

[35] Herve PL Descamps D Deloizy C Dhelft V Laubreton D Bouguyon E. Non-invasive epicutaneous vaccine against Respiratory Syncytial Virus: Preclinical proof of concept. J Control Release. (2016) 243:146–59. 10.1016/j.jconrel.2016.10.003 27720994

[36] Sarkar I Zardini Buzatto A Garg R Li L van Drunen Littel-van den Hurk S. Metabolomic and immunological profiling of respiratory syncytial virus infection after intranasal immunization with a subunit vaccine candidate. J Proteome Res. (2019) 18:1145–61. 10.1021/acs.jproteome.8b00806 30706717

[37] Schneider-Ohrum K Cayatte C Bennett AS Rajani GM McTamney P Nacel K. Immunization with low doses of recombinant postfusion or prefusion respiratory syncytial virus F primes for vaccine-enhanced disease in the cotton rat model independently of the presence of a Th1-biasing (GLA-SE) or Th2-biasing (Alum) adjuvant. J Virol. (2017) 91:e02180–16. 10.1128/JVI.02180-16 28148790 PMC5375676

[38] Zhang L Durr E Galli JD Cosmi S Cejas PJ Luo B. Design and characterization of a fusion glycoprotein vaccine for Respiratory Syncytial Virus with improved stability. Vaccine. (2018) 36:8119–30. 10.1016/j.vaccine.2018.10.032 30340881

[39] Brock LG Liu X Liang B Lingemann M Liu X Herbert R. Murine pneumonia virus expressing the fusion glycoprotein of human respiratory syncytial virus from an added gene is highly attenuated and immunogenic in rhesus macaques. J Virol. (2018) 92:e00723–18. 10.1128/JVI.00723-18 29925656 PMC6096832

[40] Green CA Sande CJ Scarselli E Capone S Vitelli A Nicosia A. Novel genetically-modified chimpanzee adenovirus and MVA-vectored respiratory syncytial virus vaccine safely boosts humoral and cellular immunity in healthy older adults. J Infect. (2019) 78:382–92. 10.1016/j.jinf.2019.02.003 30742894 PMC7172982

[41] Liang B Kabatova B Kabat J Dorward DW Liu X Surman S. Effects of alterations to the CX3C motif and secreted form of human respiratory syncytial virus (RSV) G protein on immune responses to a parainfluenza virus vector expressing the RSV G protein. J Virol. (2019) 93:e02043–18. 10.1128/JVI.02043-18 30651356 PMC6430528

[42] Lu X Yang J Wu H Yang Z Jin C Wang J. High-throughput sequencing identifies HIV-1-replication- and latency-related miRNAs in CD4(+) T cell lines. Arch Virol. (2017) 162:1933–42. 10.1007/s00705-017-3305-5 28303346

[43] Phan SI Adam CM Chen Z Citron M Liang X Espeseth AS. Genetic stability of parainfluenza virus 5-vectored human respiratory syncytial virus vaccine candidates after *in vitro* and *in vivo* passage. J Virol. (2017) 91:e00559–17. 10.1128/JVI.00559-17 28747497 PMC5599752

[44] Phan SI Zengel JR Wei H Li Z Wang D He B. Parainfluenza virus 5 expressing wild-type or prefusion respiratory syncytial virus (RSV) fusion protein protects mice and cotton rats from RSV challenge. J Virol. (2017) 91:e00560–17. 10.1128/JVI.00560-17 28747496 PMC5599740

[45] Wang D Phan S DiStefano DJ Citron MP Callahan CL Indrawati L. A single-dose recombinant parainfluenza virus 5-vectored vaccine expressing respiratory syncytial virus (RSV) F or G protein protected cotton rats and African green monkeys from RSV challenge. J Virol. (2017) 91:e00066–17. 10.1128/JVI.00066-17 28298602 PMC5432884

[46] Wiegand MA Gori-Savellini G Gandolfo C Papa G Kaufmann C Felder E. A Respiratory syncytial virus vaccine vectored by a stable chimeric and replication-deficient sendai virus protects mice without inducing enhanced disease. J Virol. (2017) 91:e02298–16. 10.1128/JVI.02298-16 28250126 PMC5411584

[47] Harcourt JL Anderson LJ Sullender W Tripp RA. Pulmonary delivery of respiratory syncytial virus DNA vaccines using macroaggregated albumin particles. Vaccine. (2004) 22:2248–60. 10.1016/j.vaccine.2003.11.050 15149784

[48] Murawski MR McGinnes LW Finberg RW Kurt-Jones EA Massare MJ Smith G. Newcastle disease virus-like particles containing respiratory syncytial virus G protein induced protection in BALB/c mice, with no evidence of immunopathology. J Virol. (2010) 84:1110–23. 10.1128/JVI.01709-09 19889768 PMC2798376

[49] Schmidt MR McGinnes LW Kenward SA Willems KN Woodland RT Morrison TG. Long-term and memory immune responses in mice against Newcastle disease virus-like particles containing respiratory syncytial virus glycoprotein ectodomains. J Virol. (2012) 86:11654–62. 10.1128/JVI.01510-12 22896618 PMC3486317

[50] Rigter A Widjaja I Versantvoort H Coenjaerts FE van Roosmalen M Leenhouts K. A protective and safe intranasal RSV vaccine based on a recombinant prefusion-like form of the F protein bound to bacterium-like particles. PLoS ONE. (2013) 8:e71072. 10.1371/journal.pone.0071072 23951084 PMC3741363

[51] Van Braeckel-Budimir N Haijema BJ Leenhouts K Bacterium-like particles for efficient immune stimulation of existing vaccines and new subunit vaccines in mucosal applications. Front Immunol. (2013) 4:282 10.3389/fimmu.2013.00282 24062748 PMC3775300

[52] Lee S Quan FS Kwon Y Sakamoto K Kang SM Compans RW Additive protection induced by mixed virus-like particles presenting respiratory syncytial virus fusion or attachment glycoproteins. Antiviral Res. (2014) 111:129–35. 10.1016/j.antiviral.2014.09.005 25239522 PMC4252885

[53] McGinnes Cullen L Schmidt MR Kenward SA Woodland RT Morrison TG. Murine immune responses to virus-like particle-associated pre- and postfusion forms of the respiratory syncytial virus F protein. J Virol. (2015) 89:6835–47. 10.1128/JVI.00384-15 25903340 PMC4468467

[54] Jorquera PA Tripp RA. Synthetic biodegradable microparticle and nanoparticle vaccines against the respiratory syncytial virus. Vaccines. (2016) 4:E45. 10.3390/vaccines4040045 27918420 PMC5192365

[55] Schwarz B Morabito KM Ruckwardt TJ Patterson DP Avera J Miettinen HM. Viruslike particles encapsidating respiratory syncytial virus M and M2 proteins induce robust T cell responses. ACS Biomater Sci Eng. (2016) 2:2324–32. 10.1021/acsbiomaterials.6b00532 29367948 PMC5777520

[56] Hwang HS Kim KH Lee Y Lee YT Ko EJ Park S. Virus-like particle vaccines containing F or F and G proteins confer protection against respiratory syncytial virus without pulmonary inflammation in cotton rats. Hum Vaccin Immunother. (2017) 13:1031–9. 10.1080/21645515.2016.1272743 28129031 PMC5443399

[57] Kim AR Lee DH Lee SH Rubino I Choi HJ Quan FS. Protection induced by virus-like particle vaccine containing tandem repeat gene of respiratory syncytial virus G protein. PLoS ONE. (2018) 13:e0191277. 10.1371/journal.pone.0191277 29338045 PMC5770062

[58] Smith TRF Schultheis K Morrow MP Kraynyak KA McCoy JR Yim KC. Development of an intradermal DNA vaccine delivery strategy to achieve single-dose immunity against respiratory syncytial virus. Vaccine. (2017) 35:2840–7. 10.1016/j.vaccine.2017.04.008 28413132 PMC5814302

[59] Smith TRF Schultheis K Broderick KE. Nucleic acid-based vaccines targeting respiratory syncytial virus: delivering the goods. Hum Vaccin Immunother. (2017) 13:2626–9. 10.1080/21645515.2017.1363134 28881156 PMC5703370

[60] Hua Y Jiao YY Ma Y Peng XL Fu YH Zhang XJ. Enhanced humoral and CD8+ T cell immunity in mice vaccinated by DNA vaccine against human respiratory syncytial virus through targeting the encoded F protein to dendritic cells. Int Immunopharmacol. (2017) 46:62–9. 10.1016/j.intimp.2017.02.023 28259002

[61] Hua Y Jiao YY Ma Y Peng XL Fu YH Zheng YP. DNA vaccine encoding central conserved region of G protein induces Th1 predominant immune response and protection from RSV infection in mice. Immunol Lett. (2016) 179:95–101. 10.1016/j.imlet.2016.09.011 27688078

[62] Eroglu E Singh A Bawage S Tiwari PM Vig K Pillai SR. Immunogenicity of RSV F DNA vaccine in BALB/c mice. Adv Virol. (2016) 2016:7971847. 10.1155/2016/7971847 27688769 PMC5027326

[63] Wu H Dennis VA Pillai SR Singh SR. RSV fusion (F) protein DNA vaccine provides partial protection against viral infection. Virus Res. (2009) 145:39–47. 10.1016/j.virusres.2009.06.012 19540885 PMC4062878

[64] Barichievy S Bakre A Host-encoded miRNAs involved in host-pathogen interactions. In: Tripp RA Karpilow J, editors. Frontiers in RNAi. Vol. 1 Oak Park, IL: Bentham Science Publishers (2014). p. 107–43. 10.2174/9781608059409114010010

[65] Mgbemena V Segovia J Chang T Bose S. Kruppel-like factor 6 regulates transforming growth factor-beta gene expression during human respiratory syncytial virus infection. Virol J. (2011) 8:409. 10.1186/1743-422X-8-409 21849067 PMC3170303

[66] Jadidi-Niaragh F Mirshafiey A. The deviated balance between regulatory T cell and Th17 in autoimmunity. Immunopharmacol Immunotoxicol. (2012) 34:727–39. 10.3109/08923973.2011.619987 22316060

[67] Mukherjee M Nair P. Autoimmune responses in severe asthma. Allergy Asthma Immunol Res. (2018) 10:428–47. 10.4168/aair.2018.10.5.428 30088364 PMC6082822

[68] Arenas-Padilla M Mata-Haro V. Regulation of TLR signaling pathways by microRNAs: implications in inflammatory diseases. Central Eur J Immunol. (2018) 43:482–9. 10.5114/ceji.2018.81351 30799997 PMC6384427

[69] Salvi V Gianello V Tiberio L Sozzani S Bosisio D. Cytokine targeting by miRNAs in autoimmune diseases. Front Immunol. (2019) 10:15. 10.3389/fimmu.2019.00015 30761124 PMC6361839

[70] Yan L Liang M Hou X Zhang Y Zhang H Guo Z. The role of microRNA-16 in the pathogenesis of autoimmune diseases: a comprehensive review. Biomed Pharmacother. (2019) 112:108583. 10.1016/j.biopha.2019.01.044 30780103

[71] Eyileten C Wicik Z De Rosa S Mirowska-Guzel D Soplinska A Indolfi C. MicroRNAs as diagnostic and prognostic biomarkers in ischemic stroke-a comprehensive review and bioinformatic analysis. Cells. (2018) 7:E249. 10.3390/cells7120249 30563269 PMC6316722

[72] Huang X Zhu Z Guo X Kong X. The roles of microRNAs in the pathogenesis of chronic obstructive pulmonary disease. Int Immunopharmacol. (2019) 67:335–47. 10.1016/j.intimp.2018.12.013 30578969

[73] Lorente-Cebrian S Gonzalez-Muniesa P Milagro FI Martinez JA. MicroRNAs and other non-coding RNAs in adipose tissue and obesity: emerging roles as biomarkers and therapeutic targets. Clin Sci. (2019) 133:23–40. 10.1042/CS20180890 30606812

[74] Pockar S Globocnik Petrovic M Peterlin B Vidovic Valentincic N. MiRNA as biomarker for uveitis - a systematic review of the literature. Gene. (2019) 696:162–75. 10.1016/j.gene.2019.02.004 30763668

[75] Amaral AJ Andrade J Foxall RB Matoso P Matos AM Soares RS. miRNA profiling of human naive CD4 T cells links miR-34c-5p to cell activation and HIV replication. EMBO J. (2017) 36:346–60. 10.15252/embj.201694335 27993935 PMC5286376

[76] Gutierrez-Vazquez C Rodriguez-Galan A Fernandez-Alfara M Mittelbrunn M Sanchez-Cabo F Martinez-Herrera DJ. miRNA profiling during antigen-dependent T cell activation: a role for miR-132-3p. Sci Rep. (2017) 7:3508. 10.1038/s41598-017-03689-7 28615644 PMC5471249

[77] Torri A Carpi D Bulgheroni E Crosti MC Moro M Gruarin P. Extracellular MicroRNA signature of human helper T cell subsets in health and autoimmunity. J Biol Chem. (2017) 292:2903–15. 10.1074/jbc.M116.769893 28077577 PMC5314185

[78] Zhou H Wu L. The development and function of dendritic cell populations and their regulation by miRNAs. Protein Cell. (2017) 8:501–13. 10.1007/s13238-017-0398-2 28364278 PMC5498339

[79] Johansson K Malmhall C Ramos-Ramirez P Radinger M. MicroRNA-155 is a critical regulator of type 2 innate lymphoid cells and IL-33 signaling in experimental models of allergic airway inflammation. J Allergy Clin Immunol. (2017) 139:1007–16.e9. 10.1016/j.jaci.2016.06.035 27492144

[80] Kim RY Horvat JC Pinkerton JW Starkey MR Essilfie AT Mayall JR. MicroRNA-21 drives severe, steroid-insensitive experimental asthma by amplifying phosphoinositide 3-kinase-mediated suppression of histone deacetylase 2. J Allergy Clin Immunol. (2017) 139:519–32. 10.1016/j.jaci.2016.04.038 27448447

[81] Lu S Mukkada VA Mangray S Cleveland K Shillingford N Schorl C. MicroRNA profiling in mucosal biopsies of eosinophilic esophagitis patients pre and post treatment with steroids and relationship with mRNA targets. PLoS ONE. (2012) 7:e40676. 10.1371/journal.pone.0040676 22815788 PMC3398046

[82] Lu TX Sherrill JD Wen T Plassard AJ Besse JA Abonia JP. MicroRNA signature in patients with eosinophilic esophagitis, reversibility with glucocorticoids, and assessment as disease biomarkers. J Allergy Clin Immunol. (2012) 129:1064–75.e9. 10.1016/j.jaci.2012.01.060 22391115 PMC3466056

[83] Malmhall C Johansson K Winkler C Alawieh S Ekerljung L Radinger M. Altered miR-155 expression in allergic asthmatic airways. Scand J Immunol. (2017) 85:300–7. 10.1111/sji.12535 28199728

[84] Yang M Eyers F Xiang Y Guo M Young IG Rosenberg HF. Expression profiling of differentiating eosinophils in bone marrow cultures predicts functional links between microRNAs and their target mRNAs. PLoS ONE. (2014) 9:e97537. 10.1371/journal.pone.0097537 24824797 PMC4019607

[85] Bartel DP. MicroRNAs: target recognition and regulatory functions. Cell. (2009) 136:215–33. 10.1016/j.cell.2009.01.002 19167326 PMC3794896

[86] Cloonan N. Re-thinking miRNA-mRNA interactions: intertwining issues confound target discovery. Bioessays. (2015) 37:379–88. 10.1002/bies.201400191 25683051 PMC4671252

[87] Fabian MR Sonenberg N Filipowicz W. Regulation of mRNA translation and stability by microRNAs. Annu Rev Biochem. (2010) 79:351–79. 10.1146/annurev-biochem-060308-103103 20533884

[88] Friedman RC Farh KK Burge CB Bartel DP. Most mammalian mRNAs are conserved targets of microRNAs. Genome Res. (2009) 19:92–105. 10.1101/gr.082701.108 18955434 PMC2612969

[89] Jia S Zhai H Zhao M. MicroRNAs regulate immune system via multiple targets. Discov Med. (2014) 18:237–47. 25425464

[90] Jost D Nowojewski A Levine E. Small RNA biology is systems biology. BMB Rep. (2011) 44:11–21. 10.5483/BMBRep.2011.44.1.11 21266101

[91] Place RF Li LC Pookot D Noonan EJ Dahiya R. MicroRNA-373 induces expression of genes with complementary promoter sequences. Proc Natl Acad Sci USA. (2008) 105:1608–13. 10.1073/pnas.0707594105 18227514 PMC2234192

[92] Huang V Place RF Portnoy V Wang J Qi Z Jia Z. Upregulation of Cyclin B1 by miRNA and its implications in cancer. Nucleic Acids Res. (2012) 40:1695–707. 10.1093/nar/gkr934 22053081 PMC3287204

[93] Bellingham SA Hill AF. Analysis of miRNA signatures in neurodegenerative prion disease. Methods Mol Biol. (2017) 1658:67–80. 10.1007/978-1-4939-7244-9_6 28861783

[94] Chandra S Vimal D Sharma D Rai V Gupta SC Chowdhuri DK. Role of miRNAs in development and disease: lessons learnt from small organisms. Life Sci. (2017) 185:8–14. 10.1016/j.lfs.2017.07.017 28728902

[95] Dahiya N Atreya CD. MicroRNAs and major blood-borne infectious viral diseases. Microrna. (2014) 2:212–8. 10.2174/2211536602666131118224225 25069445

[96] Diosa-Toro M Echavarria-Consuegra L Flipse J Fernandez GJ Kluiver J van den Berg A. MicroRNA profiling of human primary macrophages exposed to dengue virus identifies miRNA-3614-5p as antiviral and regulator of ADAR1 expression. PLoS Negl Trop Dis. (2017) 11:e0005981. 10.1371/journal.pntd.0005981 29045406 PMC5662241

[97] Kumar H Bot A. Role of MicroRNAs in shaping innate immunity and as therapeutic targets for autoimmune diseases. Int Rev Immunol. (2017) 36:123–4. 10.1080/08830185.2017.1340043 28692310

[98] Xu Z Zhou A Ni J Zhang Q Wang Y Lu J. Differential expression of miRNAs and their relation to active tuberculosis. Tuberculosis. (2015) 95:395–403. 10.1016/j.tube.2015.02.043 25936536

[99] Othumpangat S Walton C Piedimonte G. MicroRNA-221 modulates RSV replication in human bronchial epithelium by targeting NGF expression. PLoS ONE. (2012) 7:e30030. 10.1371/journal.pone.0030030 22272270 PMC3260191

[100] Bakre A Mitchell P Coleman JK Jones LP Saavedra G Teng M. Respiratory syncytial virus modifies microRNAs regulating host genes that affect virus replication. J Gen Virol. (2012) 93:2346–56. 10.1099/vir.0.044255-0 22894925 PMC3542124

[101] Thornburg NJ Hayward SL Crowe JE Jr. Respiratory syncytial virus regulates human microRNAs by using mechanisms involving beta interferon and NF-kappaB. MBio. (2012) 3:e00220–12. 10.1128/mBio.00220-12 23249809 PMC3529541

[102] Bakre A Wu W Hiscox J Spann K Teng MN Tripp RA. Human respiratory syncytial virus non-structural protein NS1 modifies miR-24 expression via transforming growth factor-beta. J Gen Virol. (2015) 96:3179–91. 10.1099/jgv.0.000261 26253191 PMC4806578

[103] Bakre AA Harcourt JL Haynes LM Anderson LJ Tripp RA. The central conserved region (CCR) of respiratory syncytial virus (RSV) G protein modulates host miRNA expression and alters the cellular response to infection. Vaccines. (2017) 5:E16. 10.3390/vaccines5030016 28671606 PMC5620547

[104] Banos-Lara MDR Zabaleta J Garai J Baddoo M Guerrero-Plata A. Comparative analysis of miRNA profile in human dendritic cells infected with respiratory syncytial virus and human metapneumovirus. BMC Res Notes. (2018) 11:432. 10.1186/s13104-018-3541-0 29970194 PMC6029031

[105] Eilam-Frenkel B Naaman H Brkic G Veksler-Lublinsky I Rall G Shemer-Avni Y. MicroRNA 146-5p, miR-let-7c-5p, miR-221 and miR-345-5p are differentially expressed in Respiratory Syncytial Virus (RSV) persistently infected HEp-2 cells. Virus Res. (2018) 251:34–9. 10.1016/j.virusres.2018.05.006 29733865

[106] Hasegawa K Perez-Losada M Hoptay CE Epstein S Mansbach JM Teach SJ. RSV vs. rhinovirus bronchiolitis: difference in nasal airway microRNA profiles and NFkappaB signaling. Pediatr Res. (2018) 83:606–14. 10.1038/pr.2017.309 29244796 PMC6174252

[107] Inchley CS Sonerud T Fjaerli HO Nakstad B. Nasal mucosal microRNA expression in children with respiratory syncytial virus infection. BMC Infect Dis. (2015) 15:150. 10.1186/s12879-015-0878-z 25884957 PMC4387708

[108] McCaskill JL Ressel S Alber A Redford J Power UF Schwarze J. Broad-spectrum inhibition of respiratory virus infection by MicroRNA mimics targeting p38 MAPK signaling. Mol Ther Nucleic Acids. (2017) 7:256–66. 10.1016/j.omtn.2017.03.008 28624201 PMC5415959

[109] Wang S Liu P Yang P Zheng J Zhao D. Peripheral blood microRNAs expression is associated with infant respiratory syncytial virus infection. Oncotarget. (2017) 8:96627–35. 10.18632/oncotarget.19364 29228557 PMC5722509

[110] Feldman SA Audet S Beeler JA. The fusion glycoprotein of human respiratory syncytial virus facilitates virus attachment and infectivity via an interaction with cellular heparan sulfate. J Virol. (2000) 74:6442–7. 10.1128/JVI.74.14.6442-6447.2000 10864656 PMC112152

[111] Tripp RA Moore D Jones L Sullender W Winter J Anderson LJ. Respiratory syncytial virus G and/or SH protein alters Th1 cytokines, natural killer cells, and neutrophils responding to pulmonary infection in BALB/c mice. J Virol. (1999) 73:7099–107. 10438795 10.1128/jvi.73.9.7099-7107.1999 PMC104227

[112] Jorquera PA Choi Y Oakley KE Powell TJ Boyd JG Palath N. Nanoparticle vaccines encompassing the respiratory syncytial virus (RSV) G protein CX3C chemokine motif induce robust immunity protecting from challenge and disease. PLoS ONE. (2013) 8:e74905. 10.1371/journal.pone.0074905 24040360 PMC3769300

[113] Prince GA Curtis SJ Yim KC Porter DD. Vaccine-enhanced respiratory syncytial virus disease in cotton rats following immunization with Lot 100 or a newly prepared reference vaccine. J Gen Virol. (2001) 82:2881–8. 10.1099/0022-1317-82-12-2881 11714962

[114] Haynes LM Jones LP Barskey A Anderson LJ Tripp RA. Enhanced disease and pulmonary eosinophilia associated with formalin-inactivated respiratory syncytial virus vaccination are linked to G glycoprotein CX3C-CX3CR1 interaction and expression of substance P. J Virol. (2003) 77:9831–44. 10.1128/JVI.77.18.9831-9844.2003 12941892 PMC224581

[115] Anderson LJ. Respiratory syncytial virus vaccine development. Semin Immunol. (2013) 25:160–71. 10.1016/j.smim.2013.04.011 23778071

[116] Livak KJ Schmittgen TD Analysis of relative gene expression data using real-time quantitative PCR and the 2^−Δ*Δ*CT^ method. Methods. (2001) 25:402–8. 10.1006/meth.2001.1262 11846609

[117] Vlachos IS Zagganas K Paraskevopoulou MD Georgakilas G Karagkouni D Vergoulis T. DIANA-miRPath v3.0: deciphering microRNA function with experimental support. Nucleic Acids Res. (2015) 43:W460–6. 10.1093/nar/gkv403 25977294 PMC4489228

[118] Vlachos IS Kostoulas N Vergoulis T Georgakilas G Reczko M Maragkakis M. DIANA miRPath v.2.0: investigating the combinatorial effect of microRNAs in pathways. Nucleic Acids Res. (2012) 40:W498–504. 10.1093/nar/gks494 22649059 PMC3394305

[119] Papadopoulos GL Alexiou P Maragkakis M Reczko M Hatzigeorgiou AG. DIANA-mirPath: Integrating human and mouse microRNAs in pathways. Bioinformatics. (2009) 25:1991–3. 10.1093/bioinformatics/btp299 19435746

[120] Jorquera PA Oakley KE Powell TJ Palath N Boyd JG Tripp RA. Layer-by-layer nanoparticle vaccines carrying the G protein CX3C motif protect against RSV infection and disease. Vaccines. (2015) 3:829–49. 10.3390/vaccines3040829 26473935 PMC4693221

[121] Karron RA Buonagurio DA Georgiu AF Whitehead SS Adamus JE Clements-Mann ML Respiratory syncytial virus (RSV) SH and G proteins are not essential for viral replication *in vitro*: clinical evaluation and molecular characterization of a cold-passaged, attenuated RSV subgroup B mutant. Proc Natl Acad Sci USA. (1997) 94:13961–6. 10.1073/pnas.94.25.13961 9391135 PMC28415

[122] Crowe JE Jr Bui PT Firestone CY Connors M Elkins WR Chanock RM. Live subgroup B respiratory syncytial virus vaccines that are attenuated, genetically stable, and immunogenic in rodents and nonhuman primates. J Infect Dis. (1996) 173:829–39. 10.1093/infdis/173.4.829 8603960

[123] Gotera J Giuffrida M Mavarez A Pons H Bermudez J Maldonado M. Respiratory syncytial virus infection increases regulated on activation normal T cell expressed and secreted and monocyte chemotactic protein 1 levels in serum of patients with asthma and in human monocyte cultures. Ann Allergy Asthma Immunol. (2012) 108:316–20. 10.1016/j.anai.2012.03.006 22541401

[124] Oshansky CM Barber JP Crabtree J Tripp RA. Respiratory syncytial virus F and G proteins induce interleukin 1alpha, CC, and CXC chemokine responses by normal human bronchoepithelial cells. J Infect Dis. (2010) 201:1201–7. 10.1086/651431 20205592 PMC2839062

[125] Conti P DiGioacchino M. MCP-1 and RANTES are mediators of acute and chronic inflammation. Allergy Asthma Proc. (2001) 22:133–7. 10.2500/108854101778148737 11424873

[126] Gulyaeva LF Kushlinskiy NE. Regulatory mechanisms of microRNA expression. J Transl Med. (2016) 14:143. 10.1186/s12967-016-0893-x 27197967 PMC4873990

[127] Correia CN Nalpas NC McLoughlin KE Browne JA Gordon SV MacHugh DE. Circulating microRNAs as potential biomarkers of infectious disease. Front Immunol. (2017) 8:118. 10.3389/fimmu.2017.00118 28261201 PMC5311051

[128] da Silva AM de Araujo JN de Freitas RC Silbiger VN. Circulating MicroRNAs as potential biomarkers of atrial fibrillation. Biomed Res Int. (2017) 2017:7804763. 10.1155/2017/7804763 28349066 PMC5352861

[129] Henshall DC Hamer HM Pasterkamp RJ Goldstein DB Kjems J Prehn JHM. MicroRNAs in epilepsy: pathophysiology and clinical utility. Lancet Neurol. (2016) 15:1368–76. 10.1016/S1474-4422(16)30246-0 27839653

[130] Iljas JD Guanzon D Elfeky O Rice GE Salomon C. Review: bio-compartmentalization of microRNAs in exosomes during gestational diabetes mellitus. Placenta. (2017) 54:76–82. 10.1016/j.placenta.2016.12.002 27939101

[131] Kumar S Vijayan M Bhatti JS Reddy PH. MicroRNAs as peripheral biomarkers in aging and age-related diseases. Prog Mol Biol Transl Sci. (2017) 146:47–94. 10.1016/bs.pmbts.2016.12.013 28253991

[132] Liu X Liu X Wu Y Wu Q Wang Q Yang Z. MicroRNAs in biofluids are novel tools for bladder cancer screening. Oncotarget. (2017) 8:32370–9. 10.18632/oncotarget.16026 28423688 PMC5458291

[133] Shigeyasu K Toden S Zumwalt TJ Okugawa Y Goel A. Emerging role of MicroRNAs as liquid biopsy biomarkers in gastrointestinal cancers. Clin Cancer Res. (2017) 23:2391–9. 10.1158/1078-0432.CCR-16-1676 28143873 PMC5433899

[134] Mauri M Kirchner M Aharoni R Ciolli Mattioli C van den Bruck D Gutkovitch N. Conservation of miRNA-mediated silencing mechanisms across 600 million years of animal evolution. Nucleic Acids Res. (2017) 45:938–50. 10.1093/nar/gkw792 27604873 PMC5314787

[135] Zhao Y Song Y Yao L Song G Teng C. Circulating microRNAs: promising biomarkers involved in several cancers and other diseases. DNA Cell Biol. (2017) 36:77–94. 10.1089/dna.2016.3426 28060535

[136] Fujio K Komai T Inoue M Morita K Okamura T Yamamoto K. Revisiting the regulatory roles of the TGF-beta family of cytokines. Autoimmun Rev. (2016) 15:917–22. 10.1016/j.autrev.2016.07.007 27392504

[137] Gagliardo R Chanez P Gjomarkaj M La Grutta S Bonanno A Montalbano AM. The role of transforming growth factor-beta1 in airway inflammation of childhood asthma. Int J Immunopathol Pharmacol. (2013) 26:725–38. 10.1177/039463201302600316 24067469

[138] Ganeshan K Johnston LK Bryce PJ. TGF-beta1 limits the onset of innate lung inflammation by promoting mast cell-derived IL-6. J Immunol. (2013) 190:5731–8. 10.4049/jimmunol.1203362 23630359 PMC3725733

[139] Kang HR Cho SJ Lee CG Homer RJ Elias JA. Transforming growth factor (TGF)-beta1 stimulates pulmonary fibrosis and inflammation via a Bax-dependent, bid-activated pathway that involves matrix metalloproteinase-12. J Biol Chem. (2007) 282:7723–32. 10.1074/jbc.M610764200 17209037

[140] Li MO Flavell RA. TGF-beta: a master of all T cell trades. Cell. (2008) 134:392–404. 10.1016/j.cell.2008.07.025 18692464 PMC3677783

[141] Li MO Sanjabi S Flavell RA. Transforming growth factor-beta controls development, homeostasis, and tolerance of T cells by regulatory T cell-dependent and -independent mechanisms. Immunity. (2006) 25:455–71. 10.1016/j.immuni.2006.07.011 16973386

[142] McCann KL Imani F. Transforming growth factor beta enhances respiratory syncytial virus replication and tumor necrosis factor alpha induction in human epithelial cells. J Virol. (2007) 81:2880–6. 10.1128/JVI.02583-06 17202225 PMC1866016

[143] Oh SA Li MO. TGF-beta: guardian of T cell function. J Immunol. (2013) 191:3973–9. 10.4049/jimmunol.1301843 24098055 PMC3856438

[144] Tu E Chia PZ Chen W TGFbeta in T cell biology and tumor immunity: angel or devil? Cytokine Growth Factor Rev. (2014) 25:423–35. 10.1016/j.cytogfr.2014.07.014 25156420 PMC4182110

[145] Gibbs JD Ornoff DM Igo HA Zeng JY Imani F. Cell cycle arrest by transforming growth factor beta1 enhances replication of respiratory syncytial virus in lung epithelial cells. J Virol. (2009) 83:12424–31. 10.1128/JVI.00806-09 19759128 PMC2786720

[146] Pokharel SM Shil NK Bose S. Autophagy, TGF-beta, and SMAD-2/3 signaling regulates interferon-beta response in respiratory syncytial virus infected macrophages. Front Cell Infect Microbiol. (2016) 6:174. 10.3389/fcimb.2016.00174 28018859 PMC5149518

[147] Thornburg NJ Shepherd B Crowe JE Jr. Transforming growth factor beta is a major regulator of human neonatal immune responses following respiratory syncytial virus infection. J Virol. (2010) 84:12895–902. 10.1128/JVI.01273-10 20926560 PMC3004333

[148] Seiler F Hellberg J Lepper PM Kamyschnikow A Herr C Bischoff M. FOXO transcription factors regulate innate immune mechanisms in respiratory epithelial cells. J Immunol. (2013) 190:1603–13. 10.4049/jimmunol.1200596 23315071

[149] Ohol YM Wang Z Kemble G Duke G. Direct inhibition of cellular fatty acid synthase impairs replication of respiratory syncytial virus and other respiratory viruses. PLoS ONE. (2015) 10:e0144648. 10.1371/journal.pone.0144648 26659560 PMC4684246

[150] Kast JI McFarlane AJ Globinska A Sokolowska M Wawrzyniak P Sanak M. Respiratory syncytial virus infection influences tight junction integrity. Clin Exp Immunol. (2017) 190:351–9. 10.1111/cei.13042 28856667 PMC5680068

[151] Rezaee F DeSando SA Ivanov AI Chapman TJ Knowlden SA Beck LA. Sustained protein kinase D activation mediates respiratory syncytial virus-induced airway barrier disruption. J Virol. (2013) 87:11088–95. 10.1128/JVI.01573-13 23926335 PMC3807305

[152] Rezaee F Harford TJ Linfield DT Altawallbeh G Midura RJ Ivanov AI. cAMP-dependent activation of protein kinase A attenuates respiratory syncytial virus-induced human airway epithelial barrier disruption. PLoS ONE. (2017) 12:e0181876. 10.1371/journal.pone.0181876 28759570 PMC5536269

[153] Wong W. Hippo-sized antiviral defenses. Sci Signal. (2017) 10:eaan4043. 10.1126/scisignal.aan4043 28400535

[154] Garofalo R Mei F Espejo R Ye G Haeberle H Baron S. Respiratory syncytial virus infection of human respiratory epithelial cells up-regulates class I MHC expression through the induction of IFN-beta and IL-1 alpha. J Immunol. (1996) 157:2506–13. 8805651

[155] Konig B Streckert HJ Krusat T Konig W. Respiratory syncytial virus G-protein modulates cytokine release from human peripheral blood mononuclear cells. J Leukoc Biol. (1996) 59:403–6. 10.1002/jlb.59.3.403 8604019

[156] Krishnan S Craven M Welliver RC Ahmad N Halonen M. Differences in participation of innate and adaptive immunity to respiratory syncytial virus in adults and neonates. J Infect Dis. (2003) 188:433–9. 10.1086/376530 12870126

[157] Cheng K Rai P Plagov A Lan X Mathieson PW Saleem MA. Rapamycin-induced modulation of miRNA expression is associated with amelioration of HIV-associated nephropathy (HIVAN). Exp Cell Res. (2013) 319:2073–80. 10.1016/j.yexcr.2013.04.011 23611955 PMC3766844

[158] de Souza AP de Freitas DN Antuntes Fernandes KE D'Avila da Cunha M Antunes Fernandes JL Benetti Gassen R. Respiratory syncytial virus induces phosphorylation of mTOR at ser2448 in CD8 T cells from nasal washes of infected infants. Clin Exp Immunol. (2016) 183:248–57. 10.1111/cei.12720 26437614 PMC4711155

[159] Kumar M Sharma A Ahmad T Mabalirajan U Aich J Agrawal A. Antagonism of mmu-mir-106a attenuates asthma features in allergic murine model. J Appl Physiol. (2012) 113:459–64. 10.1152/japplphysiol.00001.2012 22700801

[160] Lu TX Rothenberg ME. Diagnostic, functional, and therapeutic roles of microRNA in allergic diseases. J Allergy Clin Immunol. (2013) 132:3–13; quiz 14. 10.1016/j.jaci.2013.04.039 23735656 PMC3737592

[161] Herbert C Collison A Siegle JS Mattes J Foster PS Kumar RK. Altered expression of microRNA in the airway wall in chronic asthma: miR-126 as a potential therapeutic target. BMC Pulm Med. (2011) 11:29. 10.1186/1471-2466-11-29 21605405 PMC3116478

[162] Knudson CJ Hartwig SM Meyerholz DK Varga SM. RSV vaccine-enhanced disease is orchestrated by the combined actions of distinct CD4 T cell subsets. PLoS Pathog. (2015) 11:e1004757. 10.1371/journal.ppat.1004757 25769044 PMC4358888

[163] Su YC Townsend D Herrero LJ Zaid A Rolph MS Gahan ME. Dual proinflammatory and antiviral properties of pulmonary eosinophils in respiratory syncytial virus vaccine-enhanced disease. J Virol. (2015) 89:1564–78. 10.1128/JVI.01536-14 25410867 PMC4300751

