# Supplementary material for: Determining Immune and miRNA Biomarkers Related to Respiratory Syncytial Virus (RSV) Vaccine Types
Source: Front Immunol. 2019 Oct 9;10:2323. doi: 10.3389/fimmu.2019.02323 (PMC6794384; doi:10.3389/fimmu.2019.02323)
Supplement: Supplementary file 1 [file Table_1.docx]

**Supplementary Table 1.**

| **Time-point** | **Vaccine groups** | **Upregulated**  **miRs** | **Downregulated**  **miRs** |
| --- | --- | --- | --- |
| **Day 7 post-prime** | GA2-MP, FI-RSV, and CP52 | Let-7a-5p, miR-142a-5p, miR-20b-5p | miR-467f |
|  | FI-RSV and GA2-MP | Let-7f-5p, miR-15a-5p, miR-98-5p | - |
|  | FI-RSV and CP52 | miR-106a-5p, miR-195a-5p, miR-30e-5p, miR-20a-5p, miR-17-5p, miR-19b-3p | miR-182-5p, miR-466j, miR-483-5p |
|  | GA2-MP | Let-7e-5p, miR-26b-5p | - |
|  | FI-RSV | Let-7d-5p, miR-326-3p, miR-331-3p, miR-16-5p, miR-103-3p, miR-30a-5p, miR-93-5p, miR-181a-5p, miR-101a-3p, miR-15b-5p, miR-15a-3p, miR-106b-5p, miR-142a-3p, miR-19a-3p, miR-30c-5p, miR-101b-3p, miR-25-3p, miR-31-5p, let-7i-5p, let-7g-5p | miR-365-3p, miR-762 |
|  | CP52 | - | miR-466f-3p, miR-467b-3p |
| **Day 14 post-prime** | GA2-MP, FI-RSV, and CP52 | - | miR-467f |
|  | GA2-MP and CP52 | miR-15a-5p, let-7e-5p, miR-181a-5p |  |
|  | GA2-MP | Let-7a-5p, let-7f-5p, miR-331-3p, miR-182-5p, miR-103-3p, miR-26b-5p, miR-346-5p, miR-142a-5p, miR-20b-5p |  |
|  | FI-RSV | - | miR-1196-5p, miR-214-3p, miR-466f-5p, miR-466g, miR-125b-5p, miR-483-5p |
|  | CP52 | miR-574-5p, miR-15a-3p, miR-1187 | miR-365-3p |

**Supplementary Table 1:** miRNAs induced by the vaccines at post-prime vaccination. Sera miR profiles of vaccinated mice (n=4/group) were evaluated at day 7 and 14 post-prime using a miR PCR array. The relative expression levels of candidate miRs selected from the PCR array analysis were validated by RT-qPCR. Values are represented as fold-change/mock (PBS vaccinated/RSV A2 challenge). miR levels were normalized by RU6B gene expression and all samples were run in duplicate. Fold-change was calculated using 2^(-ΔΔCT)^ method. Differential expression was determined using the following criteria, if the fold change was >2, the result was reported as a fold-upregulation. If the fold-change was < 0.5, the result was reported as a fold-downregulation.

**Supplementary Table 2.**

| **Time point** | **Vaccine groups** | **Upregulated**  **miRs** | **Downregulated**  **miRs** |
| --- | --- | --- | --- |
| **Day 7 post-boost** | GA2-MP, FI-RSV, and CP52 | miR-195a-5p, miR-320-3p, let-7a-5p, miR-181b-5p, miR-672-5p, let-7e-5p, miR-17-5p, let-7c-5p, miR-714, let-7d-5p, let-7f-5p, miR-574-5p, miR-182-5p, miR-16-5p, miR-467f, miR-21a-5p, miR-130b-3p, miR-1187, miR-15b-5p, miR-26b-5p, miR-20a-5p, miR-184-3p, miR-762, miR-20b-5p, miR-25-3p, let-7i-5p, let-7g-5p | - |
|  | FI-RSV and GA2-MP | miR-331-3p, miR-103-3p, miR-29a-3p, miR-30e-5p, miR-23b-3p, miR-101a-3p, miR-106b-5p, miR-142a-5p, miR-19b-3p, miR-19a-3p, miR-101b-3p, miR-30b-5p, miR-221-3p, miR-106a-5p, miR-30a-5p, miR-346-5p, miR-93-5p, miR-29b-3p, miR-466j, miR-15a-3p, miRR-15a-3p, miR-29c-3p | - |
|  | GA2-MP and CP52 | miR-223-3p, miR-669e-5p, miR-98-5p, miR-26a-5p, miR-155-5p | - |
|  | FI-RSV and CP52 | miR-483-5p, miR-1196-5p |  |
|  | GA2-MP | miR-669f-3p, miR-142a-3p | - |
|  | FI-RSV | miR-326-3p, miR-145a-3p, miR-466f-3p, miR-24-3p, miR-181a-5p, miR-27a-3p, miR-125b-5p, miR-31-5p, miR-214-3p, miR-466f-5p, miR-365-3p, miR-146b-5p, miR-30c-5p, miR-466h-5p, miR-126a-3p | - |
| **Day 14 post-boost** | GA2-MP and FI-RSV | miR-184-3p (FI-RSV) | miR-184-3p (GA2-MP) |
|  | GA2-MP and CP52 | mir-714 (RSV CP52) | miR-714 (GA2-MP) |
|  | GA2-MP | - | miR-466g, mIR-98-5p, miR-467f, miR-669f-3p, miR-483-5p |
|  | FI-RSV | miR-146b-5p | Let-7f-5p |
|  | CP52 | Let-7d-5p, let-7a-5p, miR-672-5p, miR-182-5p, miR-16-5p, miR-20a-5p, miR-15a-3p, miR-106a-5p, miR-106b-5p, miR-17-5p, miR-93-5p, miR-195a-5p, miR-19b-3p, miR-20b-5p, miR-30c-5p, miR-31-5p, miR-25-3p, let-7i-5p, miR-30b-5p, let-7g-5p | - |

**Supplementary Table 2 miRs induced by the vaccines at post-boost vaccination.** Sera miR profiles of vaccinated mice (n=4/group) were evaluated at day 7 and 14 post-prime using a miR PCR array. The relative expression levels of candidate miRs selected from the PCR array analysis was confirmed by RT-qPCR. Values are represented as fold-change/mock (PBS vaccinated/RSV A2 challenge). miR levels were normalized by RU6B gene expression and all samples were run in duplicate. Fold-change was calculated using 2^(-ΔΔCT)^ method. Differential expression was determined using the following criteria, if the fold-change was >2, the result was reported as a fold-upregulation. If the fold-change was <0.5, the result was reported as a fold-downregulation.

| **Time point** | **Vaccine group(s)** | **Upregulated**  **miRNAs** | **Downregulated**  **miRNAs** |
| --- | --- | --- | --- |
| **Day 3 post-challenge** | GA2-MP and RSV CP52 | miR-467f, miR-184-3p | - |
|  | GA2-MP | miR-145a-5p,miR-346-5p, miR-146b-5p | miR-669e-5p |
|  | FI-RSV | - | miR-483-5p |
|  | CP52 | Let-7f-5p, miR-103-3p, miR-15b-5p, miR-101a-3p, miR-16-5p, miR-20a-5p, miR-106a-5p, miR-98-5p, miR-30a-5p, miR-17-5p, miR-195a-5p, miR-142a-5p, miR-181a-5p, miR-714, miR-31-5p, miR-101b-3p, miR-25-3p, let-7i-5p, miR-130b-3p | miR-182-5p |
| **Day 5 post-challenge** | GA2-MP, FI-RSV, and CP52 | Let-7f-5p, miR-103-3p, let-7a-5p, let-7e-5p, miR-26b-5p, miR-184-3p | - |
|  | GA2-MP and FI-RSV | miR-106a-5p, miR-93-5p, miR-195a-5p, miR-30e-5p, miR-15a-5p, miR-20a-5p, miR-106b-5p, miR-17-5p, miR-142a-3p, miR-20b-5p, miR-31-5p, let-7g-5p | - |
|  | GA2-MP and RSV CP52 | miR-346-5p | miR-145a-5p, miR-365-3p |
|  | FI-RSV and CP52 | miR-98-5p |  |
|  | GA2-MP | miR-16-5p, miR-30a-5p, miR-29b-3p, miR-181a-5p, miR-101a-3p, miR-15a-3p, miR-142a-5p, miR-19a-3p, miR-101b-3p, let-7i-5p | miR-182-5p, miR-483-5p, miR-27b-3p, miR-126a-3p |
|  | FI-RSV | Let-7d-5p, miR-26a-5p, miR-146b-5p, let-7c-5p, miR-30c-5p | miR-326-3p |
|  | CP52 | miR-27a-3p | miR-223-3p |

**Supplementary Table 3: miRNAs induced by vaccination at post-RSV challenge.** Sera miR profiles of vaccinated mice (n=4/group) were evaluated at days 7 and 14 post-prime using a miR PCR array. The relative expression levels of candidate miRs selected from the PCR array analysis was confirmed by RT-qPCR. Values are represented as fold-change/mock (PBS vaccinated/RSV A2 challenge). miR levels were normalized by RU6B gene expression and all samples were run in duplicate. Fold-change was calculated using 2^(-ΔΔCT)^ method. Differential expression was determined using the following criteria, if the fold-change was >2, the result was reported as a fold-upregulation. If the fold change was <0.5, the result was reported as a fold-downregulation.
